# Development of Fluorescent Chemosensors for Calcium and Lead Detection

**DOI:** 10.3390/molecules29020527

**Published:** 2024-01-21

**Authors:** Liliana J. Gomes, Mani Outis, Clara S. B. Gomes, Augusto C. Tomé, Artur J. Moro

**Affiliations:** 1 LAQV-REQUIMTE, Departamento de Química, Faculdade de Ciências e Tecnologia, Universidade Nova de Lisboa, 2829-516 Caparica, Portugal; lj.gomes@campus.fct.unl.pt (L.J.G.); m.hosseinzadeh@campus.fct.unl.pt (M.O.); clara.gomes@fct.unl.pt (C.S.B.G.); 2 LAQV-REQUIMTE, Department of Chemistry, University of Aveiro, 3810-193 Aveiro, Portugal; actome@ua.pt

**Keywords:** fluorescent chemosensors, coumarin-3-carboxamides, azacrowns, metal ion detection, lead, calcium

## Abstract

In the present work, several coumarin-3-carboxamides with different azacrown ether moieties were designed and tested as potential luminescent sensors for metal ions. The derivative containing a 1-aza-15-crown-5 as a metal chelating group was found to yield the strongest response for Ca^2+^ and Pb^2+^, exhibiting an eight- and nine-fold emission increase, respectively, while other cations induced no changes in the optical properties of the chemosensor molecule. Job’s plots revealed a 1:1 binding stoichiometry, with association constants of 4.8 × 10^4^ and 8.7 × 10^4^ M^–1^, and limits of detection of 1.21 and 8.04 µM, for Ca^2+^ and Pb^2+^, respectively. Computational studies suggest the existence of a PET quenching mechanism, which is inhibited after complexation with each of these two metals. Proton NMR experiments and X-ray crystallography suggest a contribution from the carbonyl groups in the coumarin-3-carboxamide fluorophore in the coordination sphere of the metal ion.

## 1. Introduction

**Figure 1:**
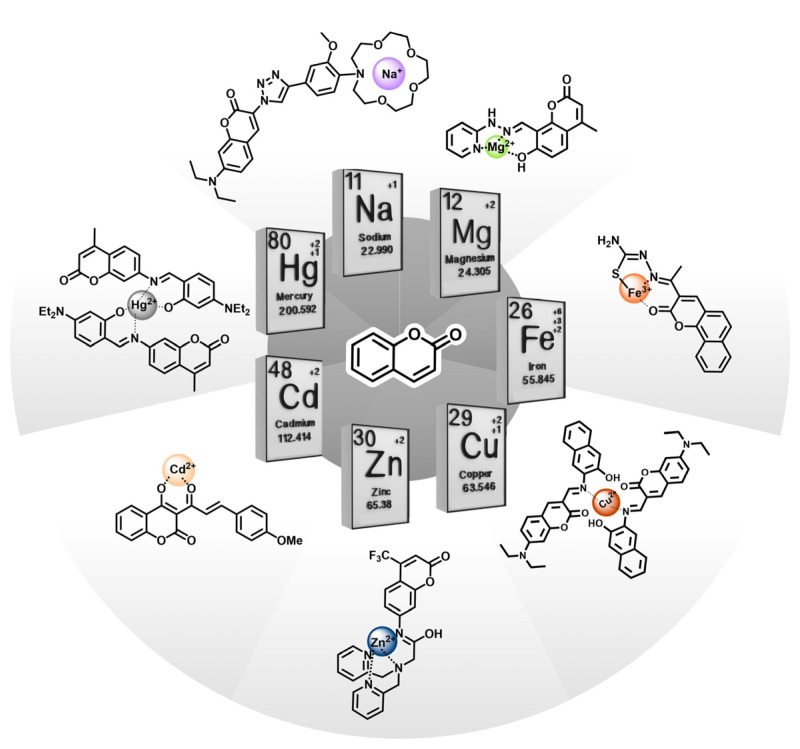
Summary of different coumarin fluorescent scaffold derivatives and their corresponding metal ions coordination: sodium [7], magnesium [14], iron [15], copper [9], zinc [16], cadmium [17] and mercury [10].

The development of sensitive and selective chemosensors capable of detecting and quantifying important analytes is key for monitoring the concentration of such chemical species in different environments and matrixes [1]. In this sense, fluorescence-based chemosensors are particularly promising given the high sensitivity of these molecules, which allows detection as low as picomolar concentrations [2]. The wide range of chromophores and fluorophores permits fine-tuning the optical properties exhibited by the final sensor molecule. A successful case is the class of coumarins, which have been used as a fluorescence scaffold for the development of chemosensors for various relevant analytes, the vast majority of which focus on metal ions [3] (Figure 1). Indeed, metal ionic species are ubiquitous in nature, and many have crucial roles for maintaining the balance of biological systems. Some of the most relevant ions include alkali (e.g., sodium and potassium), alkali-earth (e.g., calcium and magnesium) and d-block metals (e.g., iron and zinc), all of which perform multiple functions at the intracellular level [4]. On the other hand, monitoring the concentration of heavy metal ions such as lead or cadmium in water samples is of key importance, since these ions have no known biological functions and can be extremely toxic for living organisms [5].

With this in mind, we have designed and synthesized a series of coumarin-3-carboxamide derivatives bearing different chelating groups based on azacrown moieties. Azacrown ethers have been extensively used for the design of fluorescent sensors for metal species, given their capability of binding strongly to metal cations [6]. Given the hardness of oxygen heteroatoms in the azacrown, most of the examples from the literature on sensor systems involving this binding group are reported to act mainly towards sodium and potassium [7]. Nevertheless, azacrown fluorescent derivatives have also been reported for the detection of alkali-earth metals, such and Ca^2+^ [8], as well as heavy metals such Cu^2+^ [9], Hg^2+^ [10] and Pb^2+^ [11,12,13].

In the present work, the sensor molecules are based of coumarin-3-carboxamides, with a carbonyl bridging the fluorophore and the chelating unit. The designed compounds were fully characterized in terms of optical response to different metal cations, complemented by NMR studies and computational studies, to achieve a better understanding of the selectivity of the developed sensors.

## 2. Results and Discussion

### 2.1. Synthesis

**Figure 2:**
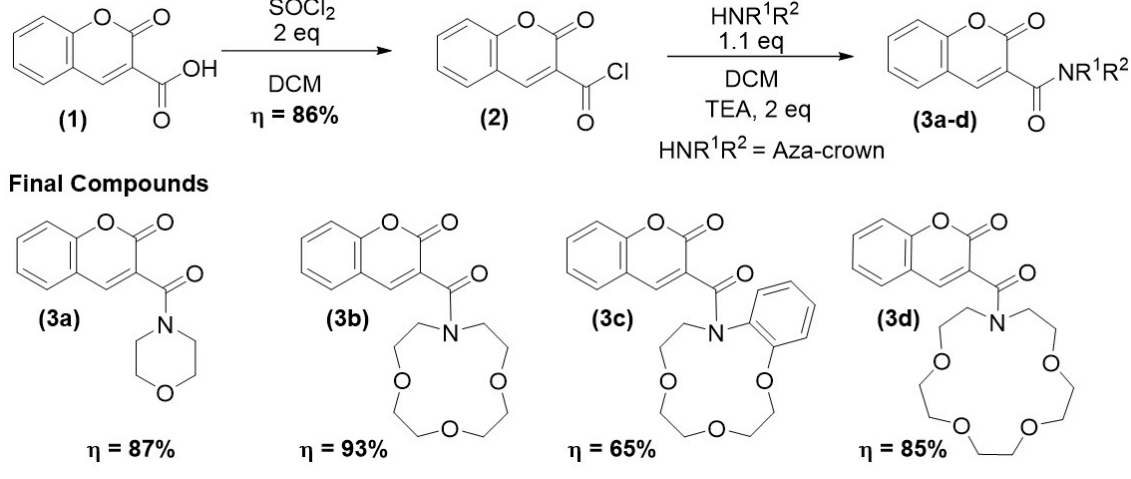
Synthetic pathway for compounds **3a-d**.

Final compounds **3a-d** were synthesized in a two-linear-step synthesis described in Figure 2. Firstly, carboxylic acid **1**, acquired commercially, was allowed to react with thionyl chloride in dichloromethane (DCM), affording acyl chloride **2** in 86% yield. Afterwards, the reaction between compound **2** and several azacrown ethers afforded the corresponding amides in 65–93% yield. The structural characterization of all compounds was carried out using NMR and mass spectrometry. A detailed analysis of the 1D and 2D NMR spectra of the final compounds led to a full structural characterization and signal attribution (see Supplementary Material).

### 2.2. UV–Vis and Fluorescence Studies

Compounds (**3a-d**) were studied through absorbance and fluorescence spectroscopies, and their general optical and photophysical characteristics are summarized in Table 1. All absorption spectra of the synthesized compounds revealed similar band shape (Figure S25), which indicates that the absorption maxima from the coumarin core remain essentially the same for all compounds, with no influence from the different ligands. Additionally, the molar extinction coefficients of the coumarin-3-carboxamides are quite similar to the one reported for coumarin (11,000 cm^−1^M^−1^) [18].

**Figure 3:**
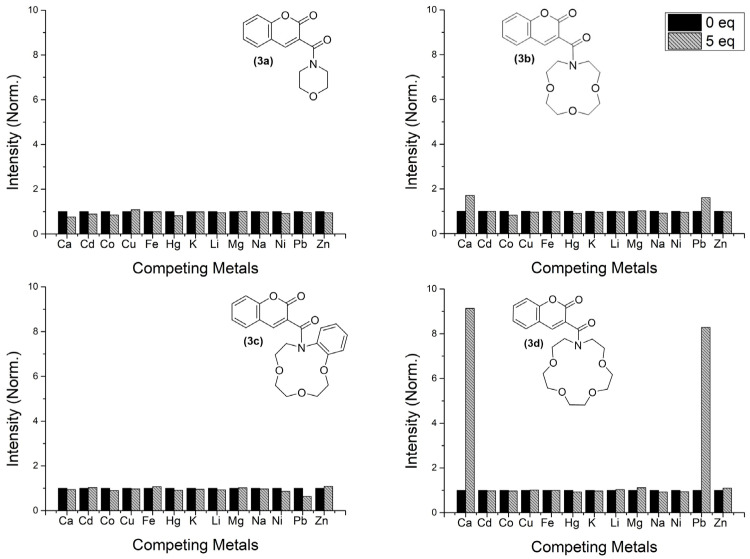
Overall response on the luminescence of compounds **3a-d** against a series of metal cations.

The synthetized coumarin-3-carboxamides have different azacrown moieties with distinctive size and rigidity. As mentioned above, azacrown ethers are reported in the literature as capable of complexing with several metal ions, depending on the size of their cavity and other features (e.g., type of heteroatoms) [19]. To further study the potential complexation between the synthesized molecules and cations, an initial screening of five equivalents of several mono- and divalent metal ions was performed for all compounds (both absorbance and emission spectra were acquired, Figure 3 and Figures S26–S29).

The conducted experiments with compound **3a** served as a control group, since no significant difference was observed in either absorption and emission spectra, which was expected due to the small size of cavity and reduced number of heteroatoms. 

Upon adding five equivalents of Ca^2+^ or Pb^2+^, compound **3b** exhibits an increase of almost twice its initial fluorescence (Φ_(3b.Ca)_ = 3.51 × 10^−4^, Φ_(3b.Pb)_ = 3.30 × 10^−4^), maintaining its original wavelength maximum at 403 nm. Unexpectedly, compound **3c** had no alteration in fluorescence or absorbance spectra after the addition of Ca^2+^. However, when **3c** is in the presence of Pb^2+^, the fluorescence is reduced by almost one-third (see Supplementary Information Figures S30 and S31 for an appropriate scale). The difference between **3b** and **3c** is the presence of an aromatic ring in the azacrown moiety, that could lead not only to a structure rigidification but also to a steric hindrance when accessing the centre of the azacrown cavity. Additionally, the ionic radius of Pb^2+^ is ca. 20% larger than Ca^2+^ [20]. These facts might indicate that calcium is more likely to be complexed on the azacrown moiety, although no effect is observed. On the other hand, lead has more probability to be both complexed in the azacrown moiety and supported by the carbonyl groups present in the coumarin structure [11,13]. As such, the fluorescence quenching observed when **3c** is exposed to lead suggests an orthogonality between the coumarin core and the azacrown moiety.

It is notable that, when comparing compound **3d** with **3b**, molecule **3d** exhibits a much stronger signal change in the presence of Ca^2+^ and Pb^2+^ since the fluorescence intensity increases nine and eight times the initial one, respectively (Φ_(3d.Ca)_ = 3.21 × 10^−3^, Φ_(3d.Pb)_ = 2.91 × 10^−4^). In this case, the higher enhanced emission on **3d** upon complexation is due to the increase of the azacrown size, which allows for a better fit in terms of “binding pocket” size. The difference in sensitivity may be related to the adopted conformation of the metal–ligand coordination sphere, resulting in a more effective inhibition of the charge-transfer quenching mechanisms from the azacrown to the fluorophore (see Section 2.3 for detailed discussion). This behaviour is in line with previously reported chemosensors for both Pb(II) and Ca(II), based on similar azacrown motifs [8,11,12,13]. All the synthesized compounds suffer negligible changes in their emission intensity (and absorption spectra) when exposed to the other metal ions (Cd^2+^, Co^2+^, Cu^2+^, Fe^2+^, Ni^2+^, Zn^2+^, Mg^2+^, Li^+^, Na^+^ and K^+^).

**Figure 4:**
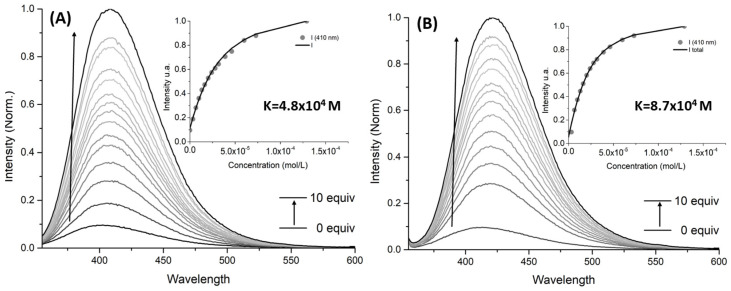
Fluorescence titration for association constant determination of complexation between compound **3d** with calcium (**A**) and lead (**B**). Conditions: variation between 0 and 10 equivalents of lead using acetonitrile as solvent, with λ_exc_ = 315 nm.

To have an insight on the sensitivity between the synthesized molecules (compound **3d**, **3c**, and **3d**) and the divalent metal ions calcium and lead, affinity constants were measured through fluorescence titration experiments (Figure 4 and Table 2).

Both compounds **3c** and **3d** exhibited luminescence enhancement in the presence of calcium and lead. However, **3d** showed a higher sensitivity for both cations than **3b**, accompanied by a slight red shift in UV absorption spectra (Figure S32). Furthermore, the affinity constant values obtained for **3d** towards both metals were about one order of magnitude higher than those obtained for **3b** (fluorescence titrations and fitting for association constants determination can be found in Figures S33–S37). These results were consistent with the larger binding cavity from **3d** (when compared to **3b**), which is able to better accommodate these two cations.

Although molecule **3c** is selective towards lead, the weak signal obtained in fluorescence ruled out this chemosensor as the best one to pursue further studies. Between compound **3b** and **3d**, molecule **3d** exhibits the strongest fluorescence signal and highest affinity towards calcium and lead. For these reasons, our focus henceforth will be on compound **3d**. 

**Figure 5:**
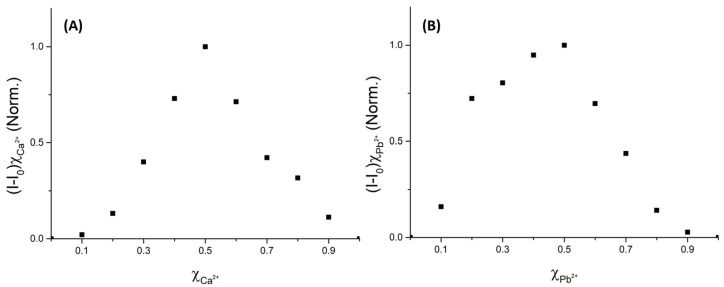
Job’s plots for **3d** in the presence of Ca^2+^ (**A**) and Pb^2+^ (**B**). For both cases, the intercept of the slope of the curves is at X_sensor_~0.5. Conditions: solvent acetonitrile, λ_exc_ = 285 nm.

Job’s plots were performed on **3d** and lead/calcium metals to confirm a stoichiometry of 1:1 (Figure 5).

**Figure 6:**
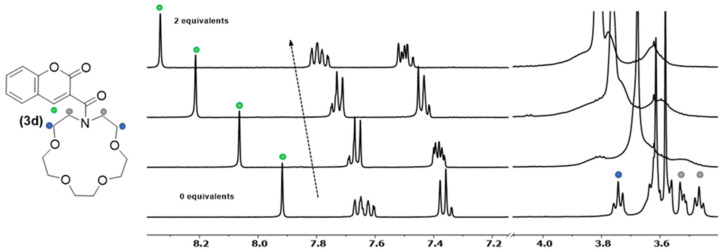
^1^H NMR titration of chemosensor **3d** with Ca^2+^ (0, 0.6, 1.5 and 2 equivalents) performed in deuterated acetonitrile.

To have an insight on where the metal complexation between **3d** and calcium occurs, an NMR titration was conducted using deuterated acetonitrile as solvent. In this experiment, ^1^H NMR spectra were acquired by varying Ca^2+^ equivalents between 0 and 2 (Figure 6). ^1^H NMR spectra results showed that the interaction between calcium and molecule **3d** first occurred in the azacrown moiety (0.5 equivalents of Ca^2+^) with the loss of resolution for the signalled peaks between 3.4 and 3.8 ppm (Figure 6), which may be related to steric/conformational constraints resulting from cation binding. 

At the same time, the singlet corresponding to proton H-4 of the coumarin nucleus (Figure 6—green dot) is continuously shifted to lower fields upon further addition of calcium. Thus, this fact supports the idea that at least one of the carbonyls is somehow also interacting with the metal.

### 2.3. Computational Studies 

**Figure 7:**
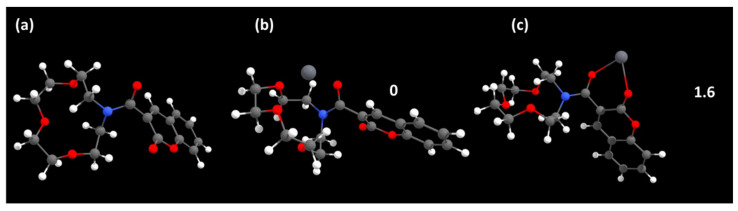
Optimized geometries of **3d** (**a**), the corresponding Pb^2+^ complex through the azacrown (**b**) and carbonyl groups (**c**). Relative energies of (**b**,**c**) are shown in kcal.mol^−1^.

To better understand the coordination mode between **3d** and the metals Pb(II)/Ca(II), the optimized geometries of the free and complexed molecules were determined (Figure 7 and Figure S38). To further explain the enhanced emission intensity on compound **3d** in the presence of a metal ion, computational studies were performed to analyze electronic states in the free molecule **3d** and the coordination products of **3d** with lead and calcium.

As expected, the thermodynamically preferred coordination mode for **3d-Pb** involves coordination of the metal centre by the azacrown moiety, with participation from the carbonyl of the amide (Figure 7b), compared, for example, with a possible coordination mode through the carbonyl groups of **3d** (Figure 7c). In the case of calcium, calculations indicate that this coordination fashion is not preferred thermodynamically (Figure S38. However, ^1^H-NMR study and the analysis of isolated single crystals by X-ray diffraction revealed that coordination through the azacrown is favoured (see section below). One possible explanation is the azacrown chelate complex (Figure S38b) is favoured kinetically. Once one coordinating group of the azacrown binds to Ca^2+^, it becomes more likely that other coordinating groups, including the adjacent carbonyl, will contribute to the coordination sphere as they are now constrained to be in close proximity and properly oriented to the metal ion. 

**Figure 8:**
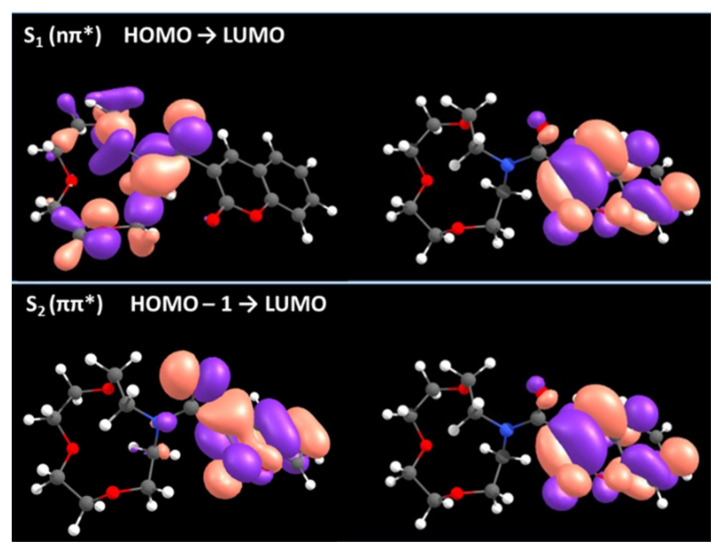
Frontier orbitals in free **3d**. The strong overlap between HOMO-1 and LUMO orbitals (**bottom**) indicates a ππ* character, while a clear charge separation is observed between HOMO and LUMO orbitals (**top**), reflecting an nπ* transition.

Additional computational studies were performed to further analyze electronic states in the free molecule **3d** and the coordination products of **3d** with calcium. Molecular orbitals involved in lowest-lying electronic transitions of **3d** were determined through TDDFT calculations and are depicted in Figure 8. Predominantly, excitation occurs from HOMO-1 to LUMO, which is essentially ππ* in nature. In contrast, HOMO to LUMO transition is essentially a dark nπ* state reflected by a very low oscillator strength (Table S5). The TDDFT simulated absorption spectrum of **3d** is in good agreement with the experimental UV-Vis in ACN (Figure S39).

According to the calculations, excitation at 315 nm corresponds essentially to a HOMO-1 → LUMO populating the S_2_ state through a local ππ* transition. HOMO orbital has a strong electron donor character, and lays 0.29 eV above HOMO-1, which suggests the occurrence of intramolecular Photoinduced Electron Transfer (PET) as the main quenching process (Figure S40).

Although the energy and nature of involved frontier orbitals are compatible with the intramolecular PET, without compelling specific experimental proofs, other dark-state quenching mechanisms cannot be ruled out. As such, we used a more general approach based on the concept of dark-state quenching, which has been previously described in the literature for a similar compound (an anthracene with an appended azacrown ether) [21]. Accordingly, to perform a dipper analysis beyond the frontier orbital energy diagram, we evaluated the electronic nature of the excited states upon relaxation on the S_1_ surface of both metal-free **3d** and coordinated to Ca^2+^ with TDDFT optimization methods. In the metal-free **3d**, assuming a fast S_2_ → S_1_ Internal Conversion (IC) according to the Kasha rule, relaxation on the S_1_ surface leads to the population of the well of a dark nπ* state. Vertical transition from the bottom of this well is characterized by the oscillation strength of almost 0 and the low energy gap of 0.83 eV (Figure S41, left). Coordination of **3d** to Ca^2+^ changes drastically the nature of the first singlet excited state to ππ*, which is populated by light excitation in the spectroscopic studies (Table S5). Relaxation on the S_1_ surface in this compound leads to the population of the well of a bright ππ* state (Figure S41, right). The calculated vertical transition energy from the bottom of the bright well (3.15 eV/394 nm) is in excellent agreement with the experimental emission energy from **3d-Ca** (3.06 eV/405 nm).

Molecular orbitals involved in the vertical transition from the calculated minimum of S_1_ are presented in Figure S42, showing the nπ* and ππ* character in metal-free **3d** and in coordinated **3d**-Ca^2+^, respectively.

### 2.4. Metal Competition 

**Figure 9:**
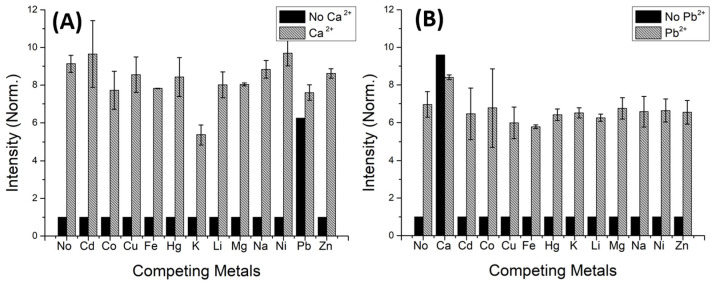
Competitive assays of chemosensor **3d** with (**A**) Ca^2+^ and (**B**) Pb^2+^ (5 equiv.), in the presence of other metal ions (5 equiv.). (λ_exc_ = 285 nm, acetonitrile).

Competitive assays were conducted using chemosensor (**3d**) in the presence of five equivalents of several metals. Afterwards, five equivalents of calcium or lead cations were added (Figure 9).

Competitive assays represented in Figure 9A suggest that the presence of lead can affect the selectivity of compound **3d** towards calcium, while other metals show little interference on the emission of **3d** (although potassium induced a smaller luminescence increase upon subsequent calcium addition). Regarding competition with lead, all assays represented in Figure 9B displayed a similar increase in emission, supporting that none of the studied metals interfered with the affinity between the chemosensor and lead, except for calcium. These results are in good agreement with the calculated affinities between **3d** and each of the two metals, and indicate some limitation in the potential use of these molecules as ion sensors. Nevertheless, one can envisage the use of **3d** for rapid luminescent screening of calcium in aqueous samples (even though the limited solubility in water suggests that **3d** should be embedded in a solid support, e.g., a polymer matrix), particularly in biological samples, where the concentration of Pb^2+^ is much lower (or null). For samples that may contain both ions, complementary methodologies (e.g., atomic absorption) might be used for full disclosure of sample assessment.

The limit of detection (LOD) was determined as 1.21 µM for **3d** towards Ca^2+^ and 8.04 µM towards Pb^2+^.

### 2.5. Complex Synthesis and X-ray Crystallography Studies

**Figure 10:**
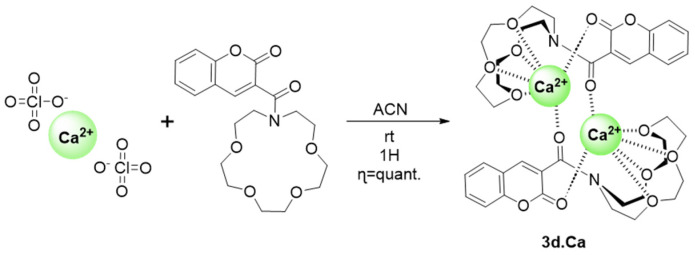
Synthesis of compound **3d.Ca**.

Prompted by the fluorescence and NMR titration studies that have yielded promising results toward the affinity/interaction between **3d** and lead/calcium metal ions, we decided to attempt the synthesis and characterization of their complexes. However, due to the toxicity of lead salts, only the synthesis of compound **3d-Ca** was conducted. Compound **3d-Ca** was achieved in quantitative yield by reacting calcium perchlorate with compound **3d**, in a 1:1 ratio, in acetonitrile for one hour at room temperature. After the semi-evaporation of the solvent, suitable crystals of **3d-Ca** were possible to isolate (Figure 10). 

^1^H NMR spectroscopy and mass spectrometry were used to characterise the reaction product. The ^1^H NMR spectrum of the complex **3d-Ca** was compared with those obtained by titration of the chemosensor **3d** with Ca^2+^ (from 0 to 2 equivalents) and they are similar. This result supports the conclusion that the same compound is formed under both conditions (Supporting Information S43 to S45).

**Figure 11:**
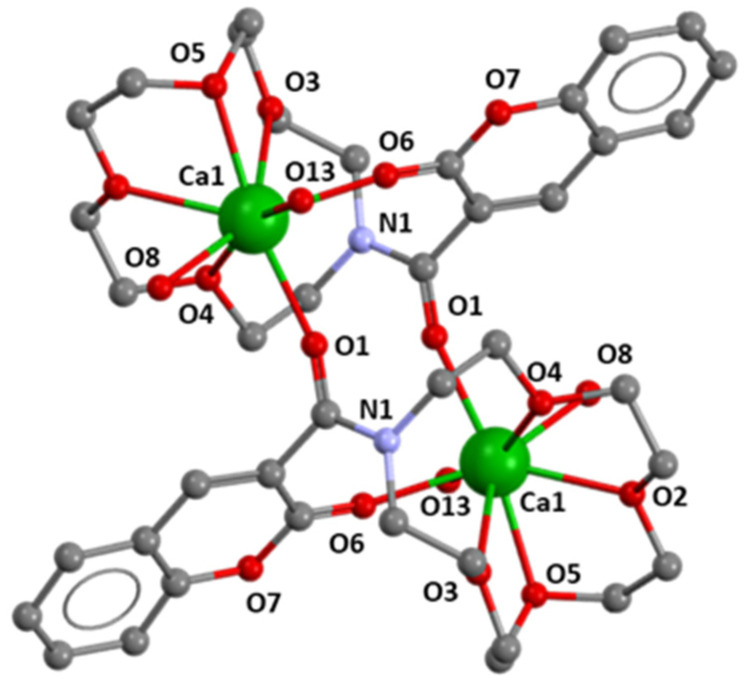
Ball and stick representation of the molecular structure of the dimeric cation of **3d.Ca**. All hydrogen atoms, four ClO_4_^–^ anions and one solvate water molecule were omitted for clarity. Atom color scheme: green – calcium, red – oxygen, blue – nitrogen, and grey – carbon.

The solid-state structure of the Ca^2+^ complex bearing ligand **3d** was determined. Compound **3d-Ca** crystalized in the triclinic crystal system, in the *P*-1 space group, with the molecular formula C_40_H_62_Ca_2_Cl_4_N_2_O_36_. Interestingly the crystal structure revealed the presence of a bimetallic cationic complex, where a half molecule is generated by the symmetry operation 1-*x*, 1-*y*, 1-*z*, associated in the form of dimers, i.e., complexes in which the ratio of metal:ligand **3d**:Ca^2+^ is 1:1, without close contact between perchlorate counterions and the cationic metal centres. The coordination sphere around each calcium ion is eight-coordinated, consisting of atoms O2, O3, O4, O5 of the azacrown unit, the O6 atom of the coumarin moiety, two water molecules and the remaining coordination position being occupied by a bridging O-atom (O1 of the coumarin fragment coordinating to the adjacent Ca centre), as shown in Figure 11 and Figure S47. The environment around the metal centre gives rise to a distorted dodecahedral geometry. In contradiction with the literature, interactions between the nitrogen atom of the azacrown macrocycle and the calcium metal ion were not found [22]. Additionally, for each metal centre, two perchlorate anions and a co-crystallized water molecule are present in the X-ray molecular structure. The bond lengths between the Ca centre and the O-donor atoms in the azacrown unit vary in the range 2.418(7) to 2.727(7) Å (Table 3). These distances are comparable with values already reported in the literature for analogous Ca-azacrown compounds [22]. In addition, the shortest distances within the coordination sphere correspond to the bonds involving the oxygen atoms belonging to the carbonyl groups of the coumarin (Ca–O6 and Ca–O1, Table 3), which can be attributed to a smaller steric hindrance and a higher availability from two lone pairs of oxygen due to geometric constraints imposed by the macrocycle. 

In the literature, the azacrown moiety is typically reported as having the ability to involve the metal cation in its centre [6,19]. In this case, it was noticeable that the metal ion was slightly out of the pocket, Figure 11 and Figure S47.

The 3D-supramolecular arrangement in complex **3d.Ca** (Figure S46) is generated by classical and non-classical hydrogen bonds between the azacrown dication and anion moieties (Figure S47 and Table S6). 

## 3. Experimental Section

### 3.1. General Information and Instruments

All used chemicals were of analytical grade and used as purchased. Fine chemicals were acquired from Sigma-Aldrich (Burlington, MA, USA) and TCI (Shanghai, China), while solvents were purchased either from Carlo Erba or Sigma-Aldrich. Thin-layer chromatography (TLC) was carried out on aluminum-backed Silica-Gel 60 F254 plates (M *DC-Fertigfolien ALUGRAM ^®^ Xtra SIL G/UV 254 nm*). Flash column chromatography was performed using Silica-Gel 60, 70–230 mesh and 230–400 mesh particle sizes as stationary phases, in the cases of regular and flash [23].

The ^1^H and ^13^C NMR (nuclear magnetic spectroscopy) spectra were acquired with a Bruker Avance III 400 (Billerica, MA, USA), at 400 and 101 MHz, respectively. 

The electrospray mass spectra were acquired on a linear ion trap mass spectrometer LXQ (ThermoFinnigan, San Jose, CA, USA). Data acquisition and analysis were performed using the Xcalibur Data System (version 2.0, ThermoFinnigan, San Jose, CA, USA). ESI conditions were as follows: electrospray voltage 5 kV in positive mode; capillary temperature was 275 °C and the sheath gas flow was 5 U.

### 3.2. Synthesis 

#### 3.2.1. Synthesis of 2-oxo-2H-Chromene-3-Carbonyl Chloride (**2**)

Coumarin-3-carboxylic acid (2.00 g, 10.52 mmol) was dissolved in dry dichloromethane (DCM) (100 mL) under an inert atmosphere and then thionyl chloride (1.5 mL, 21.04 mmol, 2 equiv.) was added dropwise at 0 °C. The reaction was controlled by TLC [dichloromethane:acetone (8:2)] and stopped after 24 H. The solvent was evaporated utilizing a rotary evaporator and dried under vacuum to afford 1.9 g of a white solid that was confirmed to be compound **2**. ^1^H NMR (400 MHz, CDCl_3_) δ 8.85 (s, 1H), 7.81–7.69 (m, 2H), 7.42 (d, *J* = 15.5 Hz, 2H).

#### 3.2.2. Synthesis of Coumarin-3-Carboxamide Derivatives (**3a-d**): General Procedure

Acyl chloride **2** (1 eq.) was added to a solution of the azacrown (**a-d**) (1.1 equiv.) and triethylamine (2 equiv.) in dry dichloromethane at room temperature. The reaction was monitored by TLC using dichloromethane:acetone (9:1) as eluent. When the reaction was considered complete, the mixture was diluted with water, neutralized to pH~7 with HCl 1 M and extracted with DCM. The organic phase was concentrated utilizing a rotary evaporator.

*3-(Morpholine-4-carbonyl)-2H-chromen-2-one* (**3a**)

No further purification was required. White powder (216 mg, 87% yield). Mp = 120.6–121.1 °C. ^1^H NMR (400 MHz, CDCl_3_) δ 7.96 (s, 1H), 7.65–7.52 (m, 2H), 7.40–7.29 (m, 2H), 3.79 (s, 4H), 3.75–3.69 (m, 2H), 3.40 (t, *J* = 4.7 Hz, 2H). ^13^C NMR (101 MHz, CDCl_3_) δ 163.70, 158.09, 154.32, 143.88, 133.17, 128.75, 125.13, 124.95, 118.40, 117.00, 66.84, 66.76, 47.78, 42.76. ESI-MS: *m*/*z* 260.1 [M + H]^+^.

*3-(1,4,7-Trioxa-10-azacyclododecane-10-carbonyl)-2H-chromen-2-one* (**3b**)

No further purification was required. White powder (308 mg, 93% yield). Mp = 134.4–135.7 °C. ^1^H NMR (400 MHz, CDCl_3_) δ 7.90 (s, 1H), 7.64–7.53 (m, 1H), 7.50 (dd, *J* = 7.8, 1.6 Hz, 1H), 7.42–7.27 (m, 2H), 3.89 (t, *J* = 4.7 Hz, 2H), 3.81 (s, 2H), 3.74–3.61 (m, 8H), 3.61–3.57 (m, 2H), 3.52 (t, *J* = 4.8 Hz, 2H). ^13^C NMR (101 MHz, CDCl_3_) δ 166.09, 158.57, 154.07, 142.68, 132.50, 128.55, 125.55, 124.88, 118.59, 116.93, 71.12, 70.60, 70.32, 69.97, 69.16, 68.97, 51.59, 48.46. ESI-MS: *m/z* 348.2 [M + H]^+^.

*3-(2,3,5,6,9,10-hexahydro-8H-benzo[h][1,4,7]trioxa[10]azacyclododecine-10-carbonyl)-2H-chromen-2-one* (**3c**)

Purified by preparative TLC using DCM:Acetone (9:1) as eluent. White powder (63 mg, 65% yield). Mp = 189.2–191.0 °C. ^1^H NMR (400 MHz, CDCl_3_) δ 7.69 (s, 1H), 7.45 (ddd, *J* = 8.5, 7.4, 1.6 Hz, 2H), 7.35 (d, *J* = 1.6 Hz, 3H), 7.22–7.10 (m, 2H), 6.90 (td, *J* = 7.6, 1.3 Hz, 1H), 6.69 (dd, *J* = 8.3, 1.3 Hz, 1H), 4.73 (ddd, *J* = 14.3, 6.6, 2.4 Hz, 1H), 4.20 (dt, *J* = 10.8, 5.0 Hz, 1H), 3.98 (ddd, *J* = 9.9, 7.1, 2.3 Hz, 1H), 3.95–3.89 (m, 2H), 3.82–3.62 (m, 4H), 3.49 (ddd, *J* = 11.6, 6.0, 3.2 Hz, 1H), 3.35 (ddd, *J* = 14.4, 7.1, 2.4 Hz, 1H). ^13^C NMR (101 MHz, CDCl_3_) δ 165.88, 157.76, 153.99, 140.82, 132.16, 131.01, 129.54, 129.50, 128.20, 126.13, 124.55, 121.31, 118.20, 116.78, 112.93, 71.96, 70.52, 69.94, 69.18, 68.30, 51.11. ESI-MS: *m/z* 396.3 [M + H]^+^.

*3-(1,4,7,10-Tetraoxa-13-azacyclopentadecane-13-carbonyl)-2H-chromen-2-one* (**3d**)

No further purification. White powder (154 mg, 85% yield). Mp = 76.6–77.7 °C. ^1^H NMR (400 MHz, CDCl_3_) δ 7.88 (s, 1H), 7.54 (td, *J* = 8.0, 1.4 Hz, 2H), 7.40–7.28 (m, 2H), 3.79 (dt, *J* = 18.8, 6.2 Hz, 6H), 3.72–3.61 (m, 10H), 3.61–3.55 (m, 2H), 3.51 (t, *J* = 6.3 Hz, 2H). ^13^C NMR (101 MHz, CDCl_3_) δ 165.86, 158.41, 154.09, 142.43, 132.60, 128.57, 125.75, 124.94, 118.55, 116.91, 71.48, 70.64, 70.47, 70.41, 70.38, 70.37, 69.72, 68.88, 51.26, 48.54. ESI-MS: *m/z* 392.3 [M + H]^+^.

*Complex 3-(1,4,7,10-Tetraoxa-13-azacyclopentadecane-13-carbonyl)-2H-chromen-2-one with calcium* (**3d.Ca**)

To a solution of 10 mL of dry acetonitrile (ACN), calcium perchlorate (164 mg, 418 µmol, 1 equiv.) and compound **3d** (100 mg, 418 µmol, 1 equiv.). After one hour of stirring, the reaction crude was semi-evaporated allowing the formation of crystals. Colourless crystals (268 mg, quantitative yield). ^1^H NMR (500 MHz, Acetonitrile-d3) δ 7.74 (s, *J* = 0.6 Hz, 1H), 7.26–7.16 (m, 2H), 6.98–6.89 (m, 2H), 3.26 (m, 21H). ^13^C NMR (126 MHz, CD3CN) δ 170.11, 162.03, 154.58, 147.57, 135.16, 130.58, 126.81, 123.68, 119.46, 117.62, 71.04, 70.22, 69.89, 55.27. ESI-MS: *m*/*z* 476.2 [M + Na]^+^.

### 3.3. UV–Vis and Fluorescence Measurements

Solutions for UV-Vis absorption and fluorescence measurements were prepared by adding an aliquot of a stock solution (compounds **3a-d**) to a quartz cell, both in acetonitrile, to achieve the desirable concentration. ([**3a**]_stock solution_ = 6.5 × 10^−4^ M; [**3b**]_stock solution_ = 1.09 × 10^−4^ M; [**3c**]_stock solution_ = 1.09 × 10^−4^ M; [**3d**]_stock solution_ = 1.09 × 10^−4^ M).

Metal ion titrations were performed in batch by adding a solution containing the metal ion and chemosensor to a cuvette containing solely the chemosensor. The limits of detection (LOD) of Ca^2+^ and Pb^2+^ were determined according to IUPAC guidelines [24], by measuring five independently prepared samples of compound **3d** with no metal (blank) and applying the formula: LOD = 3σ/*b*, where σ represents the standard deviation of these measurements, and *b* represents the slope over a fixed linear range. Absorption spectra were acquired in a 1 cm quartz cuvette on a Varian Cary 100 Bio UV-spectrophotometer. Emission spectra were obtained in a 1 cm fluorescence quartz cuvette using a Horiba-Jobin-Yvon SPEX Fluorolog 3.22 spectrofluorometer.

Fluorescence quantum yields for compounds **3a-d** were determined using 7-hydroxycoumarin (φ_f_ = 0.08, in methanol) as reference [19]. The binding constants for the synthesized molecules and metal ions were determined by fitting the experimental data to a Henderson–Hasselbalch binding model using the Solver Add-In from Microsoft Excel [25].

### 3.4. DFT Calculations

DFT calculations were carried out with the program Gaussian 16 using the B3LYP functional. The 6-31G** basis set for the light atoms and Def2TZVP triple zeta basis set and the associated effective core potential (ECP) were used for Pb. All calculations, that is, geometry optimization and TDDFT, were performed without symmetry constraints in acetonitrile, considering the solvent effect with the SMD solvation method. Molecular structures and orbitals were drawn using Chemcraft.

### 3.5. X-ray Diffraction Studies

A crystal from compound **3d-Ca** suitable for single-crystal X-ray analysis was selected, covered with Fomblin (polyfluoro ether oil) and mounted on a nylon loop. The data were collected at 293(2) K on a Bruker D8 Venture diffractometer equipped with a Photon II detector, using graphite monochromated Mo-Kα radiation (λ = 0.71073 Å). The data were processed using the APEX4 suite software package (v2022.1-1), which includes integration and scaling (SAINT), absorption corrections (SADABS) [26] and space group determination (XPREP). Structure solution and refinement were done using direct methods with the programs SHELXT 2018/2 [26,27] and SHELXL-2019/2 inbuilt in APEX and WinGX-Version 2021.3 [28] software packages. The crystals of **3d∙Ca** were of low quality and showed poor diffracting power, with diffraction spots from high angles very weak, which, consequently, led to low quality data and a high Rint. Several attempts on different crystals were performed, although they were unsuccessful in obtaining better crystal data. Nevertheless, the structure refined to convergence and the results are in agreement with the remaining analytical data. All non-hydrogen atoms were refined anisotropically and were inserted in idealized positions and allowed to refine riding on the parent carbon atom. The molecular diagrams were drawn with Mercury [29], included in the software package. Crystal data and structure refinement details are given in Tables S7–S12. The data were deposited in CCDC under the deposit number 2310426 for **3d∙Ca**.

## 4. Conclusions

We have successfully synthesized three new coumarin-3-carboxamide azacrown derivatives. Optical spectroscopy studies revealed that two of the molecules present a strong response towards calcium and lead, with a fluorescence enhancement, while a third molecule bearing a benzo-fused azacrown moiety is selective towards lead, although a fluorescence quenching is observed. Computational calculations, proton NMR and X-Ray crystallography studies on the better performing chemosensor (**3d**) indicated the contribution from ancillary carbonyl groups (from the amide and the coumarin fluorophore), which strongly increases the sensitivity and selectivity of the azacrown moiety, opening up the possibility of new molecular designs for the detection of larger cations through the use of non-covalent complementary interactions within the same sensor molecule.

## Figures and Tables

**Table 1:** Summarized photophysical characterization of coumarin-3-carboxamide derivatives in acetonitrile (ACN). ^a^ Maximum absorption wavelength; ^b^ Molar extinction coefficient at λ_max_; ^c^ fluorescence quantum yield.

Compound	λ_abs_ (nm)	λ_em_ (nm)	ε ^b^ (cm^−1^M^−1^)	Φ ^c^ (×10^−4^)
**3a**	285 ^a^, 310	408	10,908	1.62
**3b**	285 ^a^, 315	403	7501	2.05
**3c**	280 ^a^, 320	400	7973	0.361
**3d**	285 ^a^, 315	405	10,414	3.51

**Table 2:** Association constants (K) for chemosensors (**3b**), (**3c**) and (**3d**) with metals calcium and lead.

Compound	K_calcium_ (M^−1^)	K_lead_ (M^−1^)
**3b**	3.4 × 10^3^	5.3 × 10^3^
**3c**	-	2.2 × 10^4^
**3d**	4.8 × 10^4^	8.7 × 10^4^

**Table 3:** Selected bond lengths for **3d.Ca**.

Atoms	Distance (Å)	Moiety
O(2)-Ca(1)	2.475(7)	Azacrown
O(3)-Ca(1)	2.727(7)	Azacrown
O(4)-Ca(1)	2.505(6)	Azacrown
O(5)-Ca(1)	2.418(7)	Azacrown
O(6)-Ca(1)	2.369(7)	Coumarin
O(8)-Ca(1)	2.410(2)	-
O(13)-Ca(1)	2.488(2)	-
O(1) #-Ca(1) *	2.376(7)	Coumarin

* #—atom generated by the symmetry operation (1-*x*, 1-*y*, 1-*z*).

## Data Availability

The data presented in this study are available in article and Supplementary Materials.

## Supplementary Materials

The following supporting information can be downloaded at: https://www.mdpi.com/article/10.3390/molecules29020527/s1.
