# Supplementary material for: Development of Fluorescent Chemosensors for Calcium and Lead Detection
Source: Molecules. 2024 Jan 21;29(2):527. doi: 10.3390/molecules29020527 (PMC10820191; doi:10.3390/molecules29020527)
Supplement: Supplementary file 1 [file molecules-29-00527-s001.zip › molecules-2802154-supplementary.pdf]

SUPPORTING INFORMATION

Development of Fluorescent Chemosensors for Calcium and Lead Detection

Liliana J. Gomes [1], Mani Outis [1], Clara S. B. Gomes [1], Augusto C. Tomé [2] and Artur J. Moro [1,*]

[1] LAQV-REQUIMTE, Departamento de Química, Faculdade de Ciências e Tecnologia, Universidade Nova de Lisboa, 2829-516 Caparica, Portugal; lj.gomes@campus.fct.unl.pt (L.J.G.); m.hosseinzadeh@campus.fct.unl.pt (M.O.); clara.gomes@fct.unl.pt (C.S.B.G.)

[2] LAQV-REQUIMTE, Department of Chemistry, University of Aveiro, 3810-193 Aveiro, Portugal; actome@ua.pt

* Correspondence: artur.moro@fct.unl.pt

Table S1. Full NMR peak assignment for chemosensor **3a**.

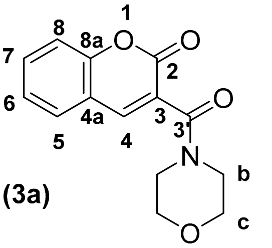

(3a)

Position	^{1}H	^{13}C
2	-	158.10
3	-	125.14
4	7.90	143.88
4a	-	118.40
5	7.50	128.75
6	7.30	124.95
7	7.56	133.17
8	7.34	117.00
8a	-	154.32
3’	-	163.70
b	3.40, 3.72	47.78, 42.76
c	3.79	66.84, 66.76

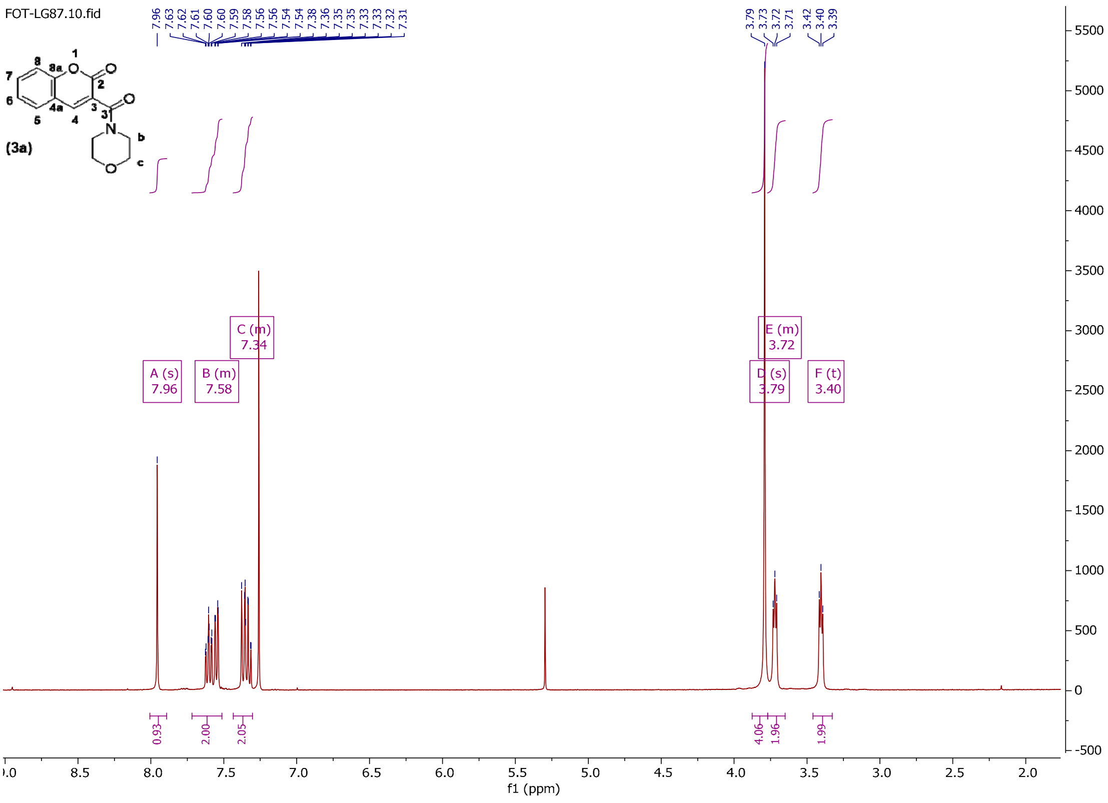

Figure S1. ^{1}H NMR spectrum of **3a**.

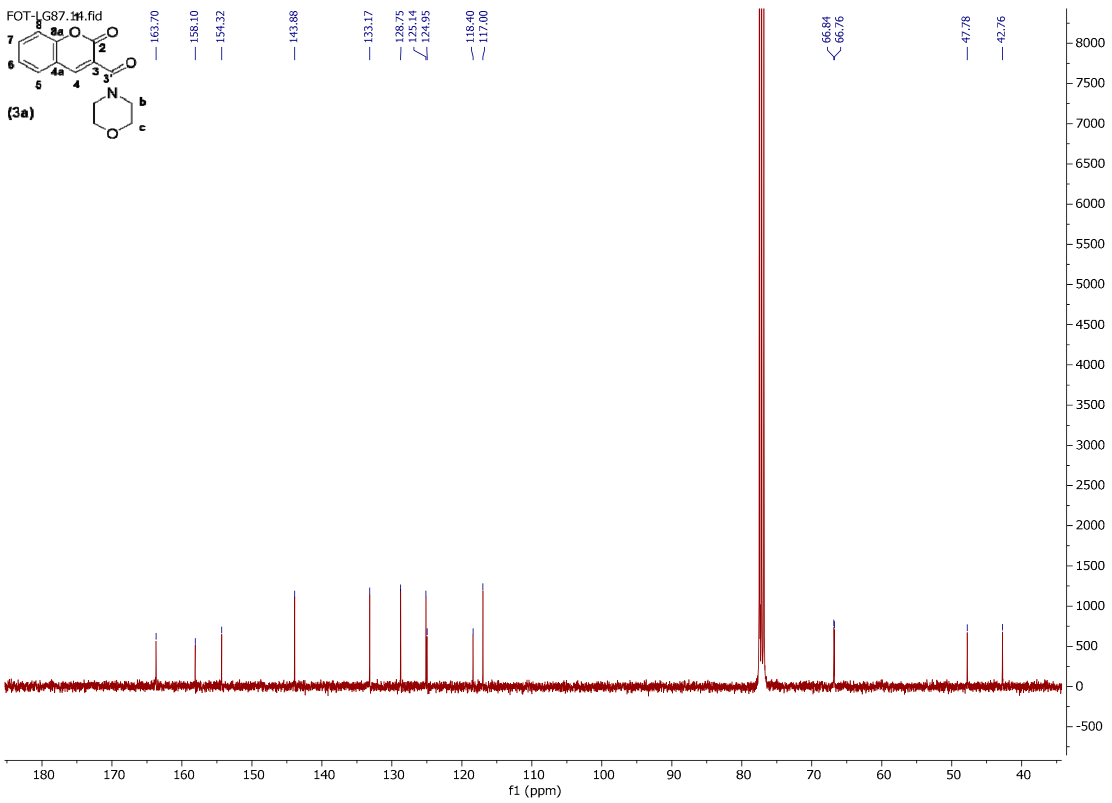

Figure S2. ^{13}C NMR spectrum of **3a**.

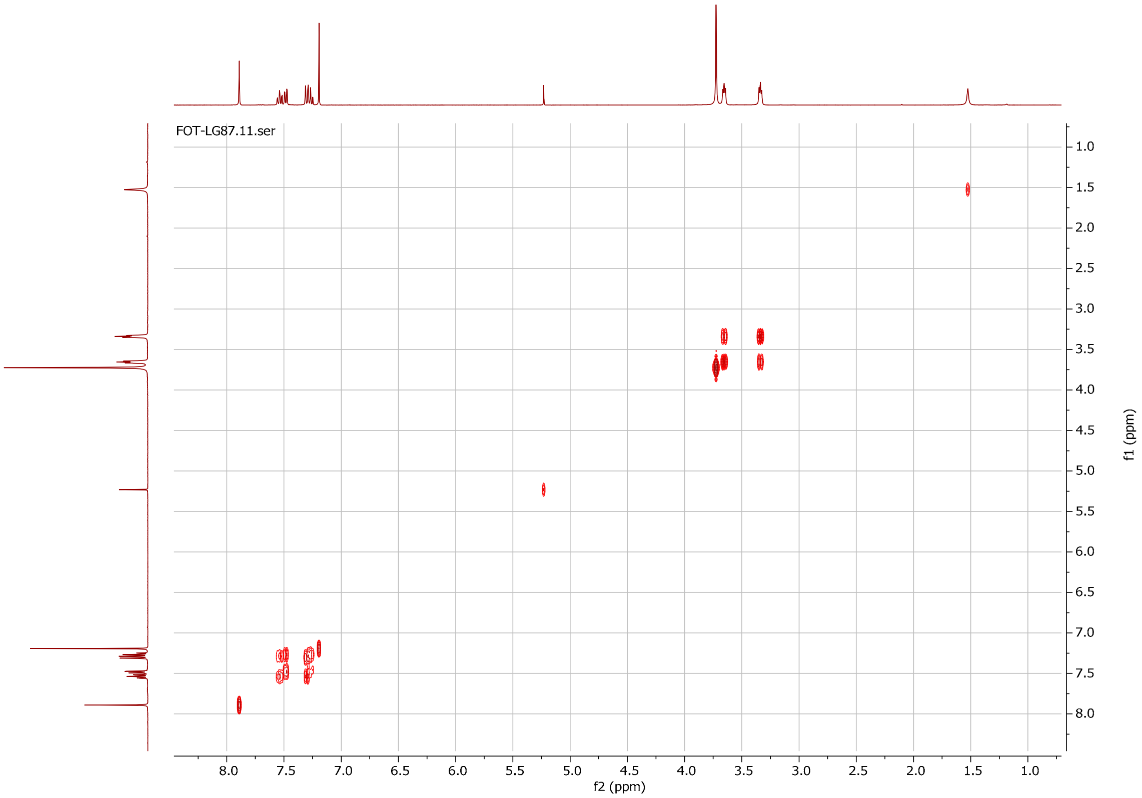

Figure S3. ^{1}H-^{1}H COSY NMR spectrum of **3a**.

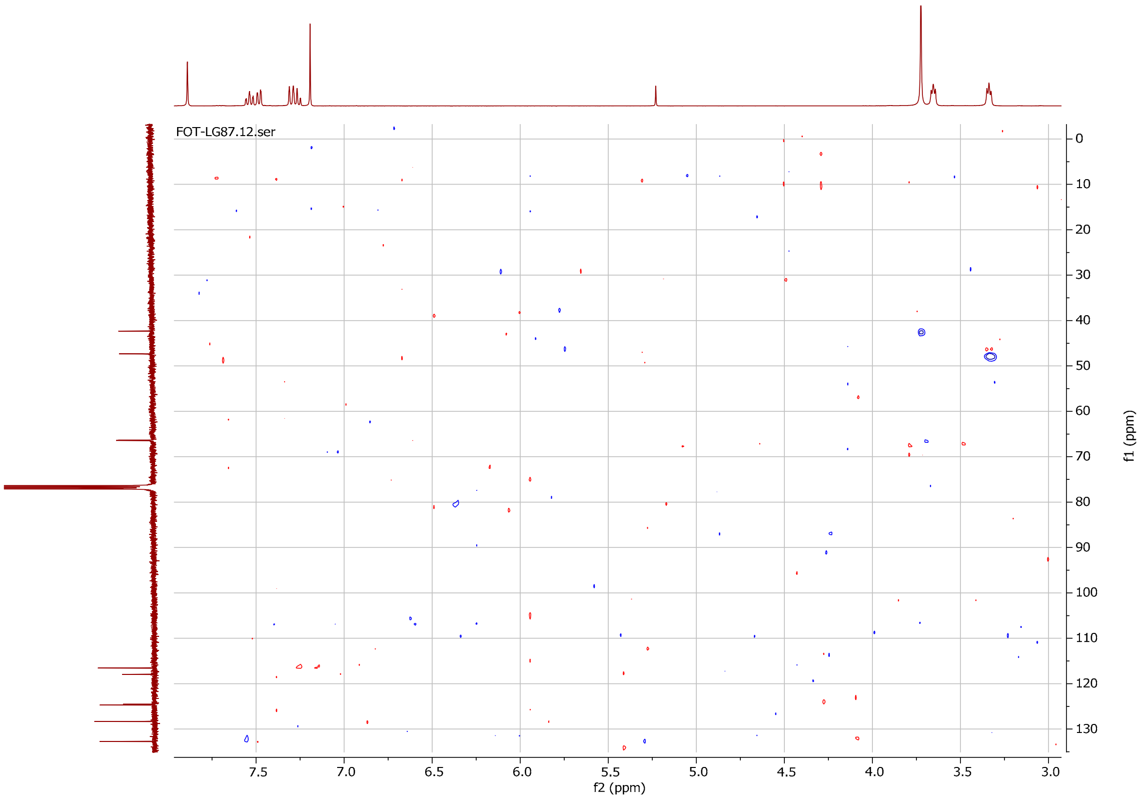

Figure S4. ^{1}H-^{13}C HSQC NMR spectrum of **3a**.

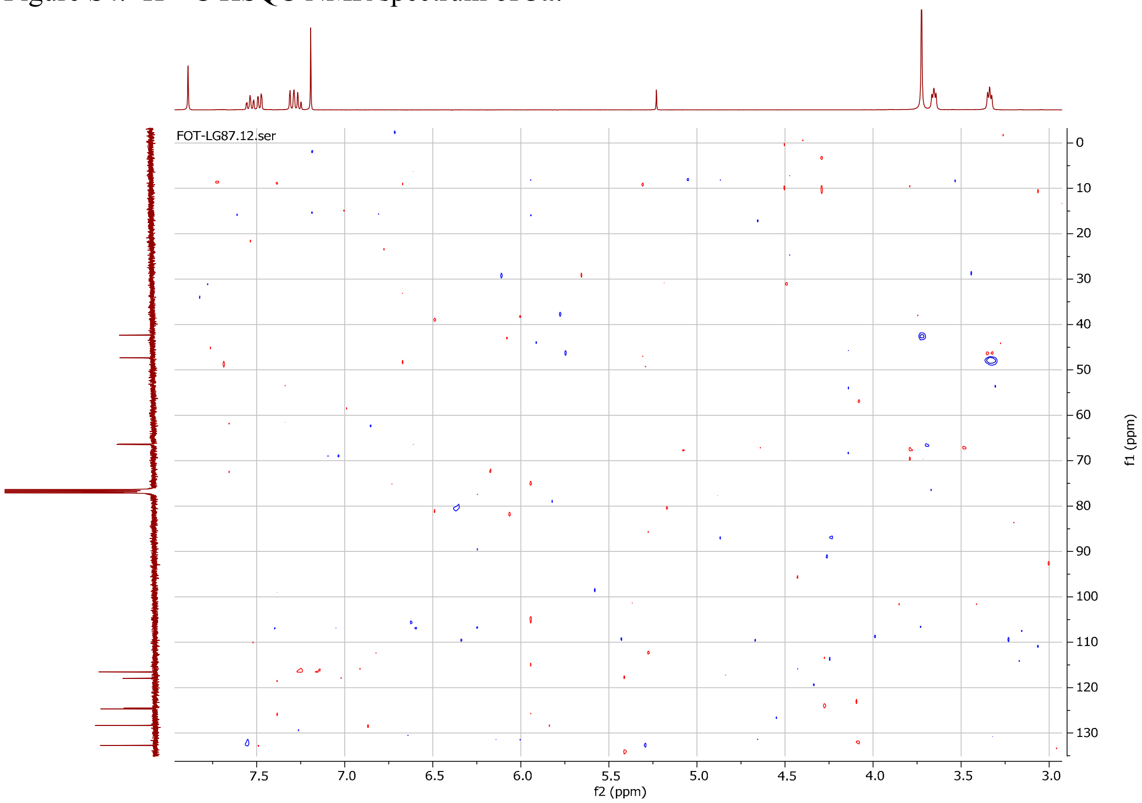

Figure S5. ^{1}H-^{13}C HMBC NMR spectrum of **3a**.

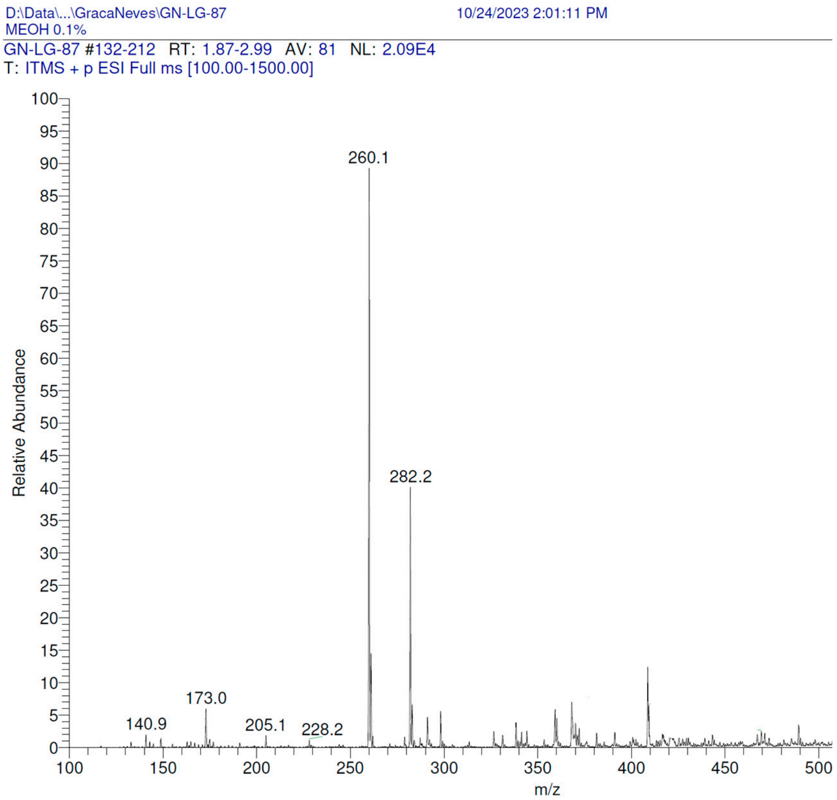

Figure S6. Mass spectrum of **3a**.

Table S2. Full NMR peak assignment for chemosensor **3b**.

Position	^{1}H	^{13}C
2	-	158.57
3	-	125.55
4	7.90	142.68
4a	-	118.59
5	7.50	128.55
6	7.30	124.88
7	7.56	132.50
8	7.34	116.93
8a	-	154.07
3’	-	166.09
b	3.89	48.46/51.59
c	3.66 – 3.62	68.97/69.16
e, f	3.52-3.81	69.19-71.12

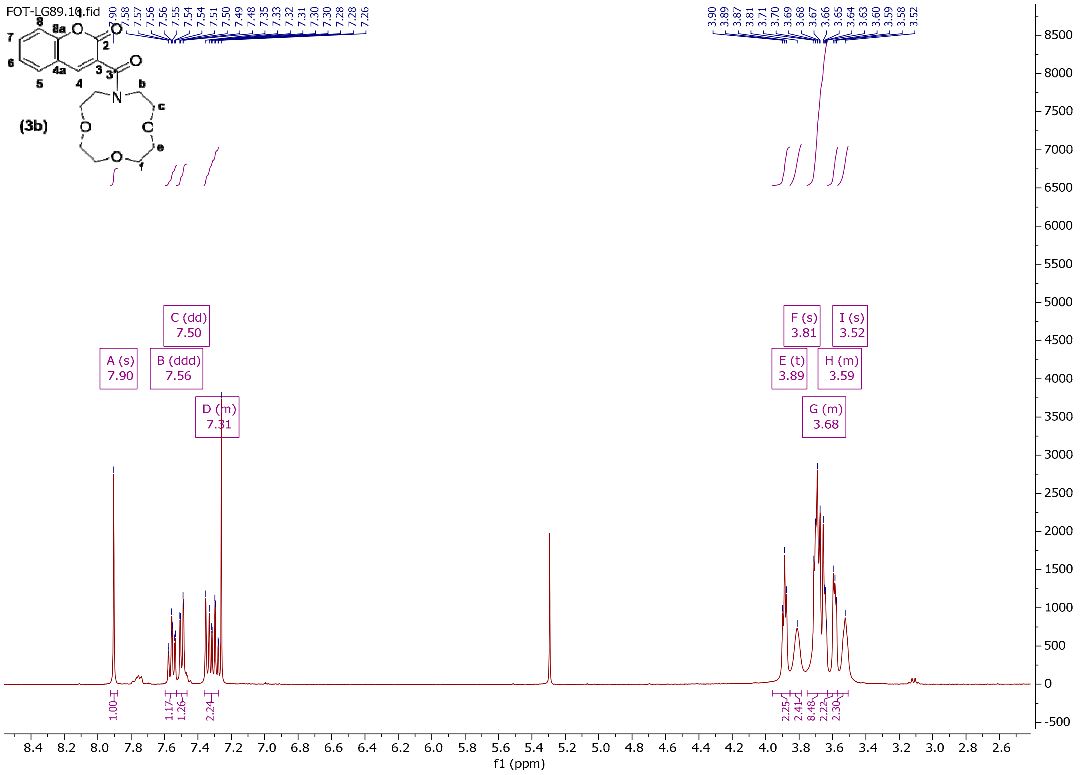

Figure S7. ^{1}H NMR spectrum of **3b**.

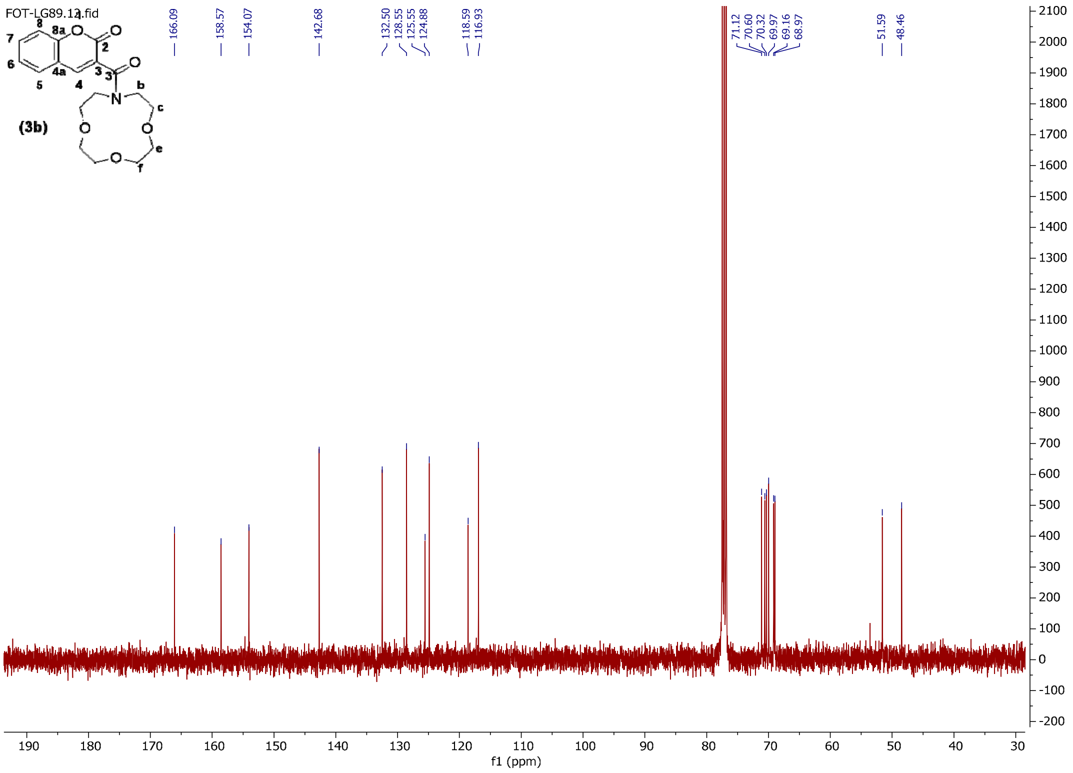

Figure S8. ^{13}C NMR spectrum of **3b**.

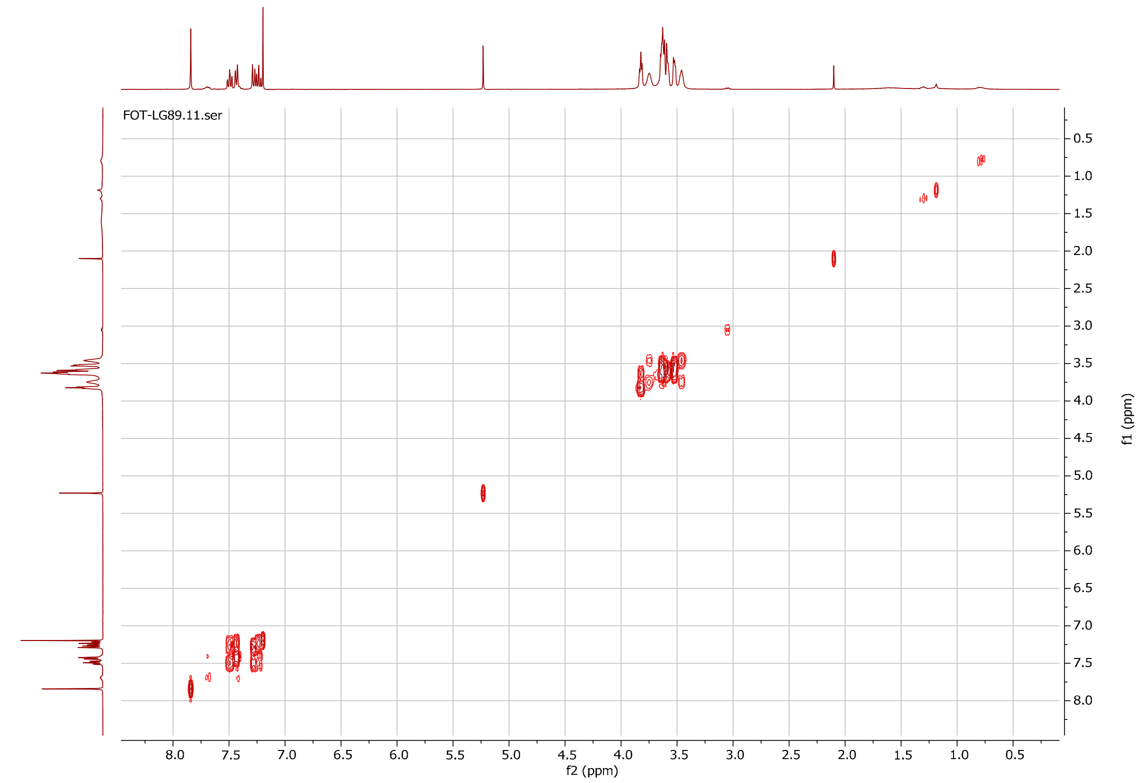

Figure S9. ^{1}H-^{1}H COSY NMR spectrum of **3b**.

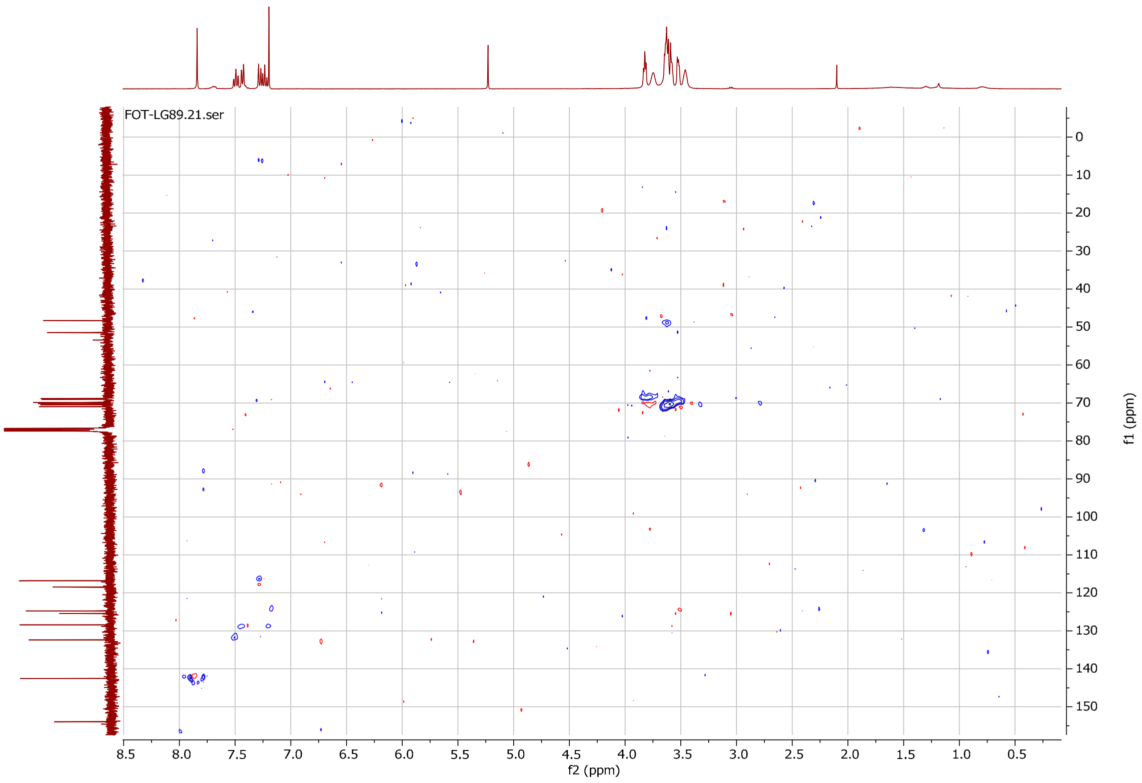

Figure S10. ^{1}H-^{13}C HSQC NMR spectrum of **3b**.

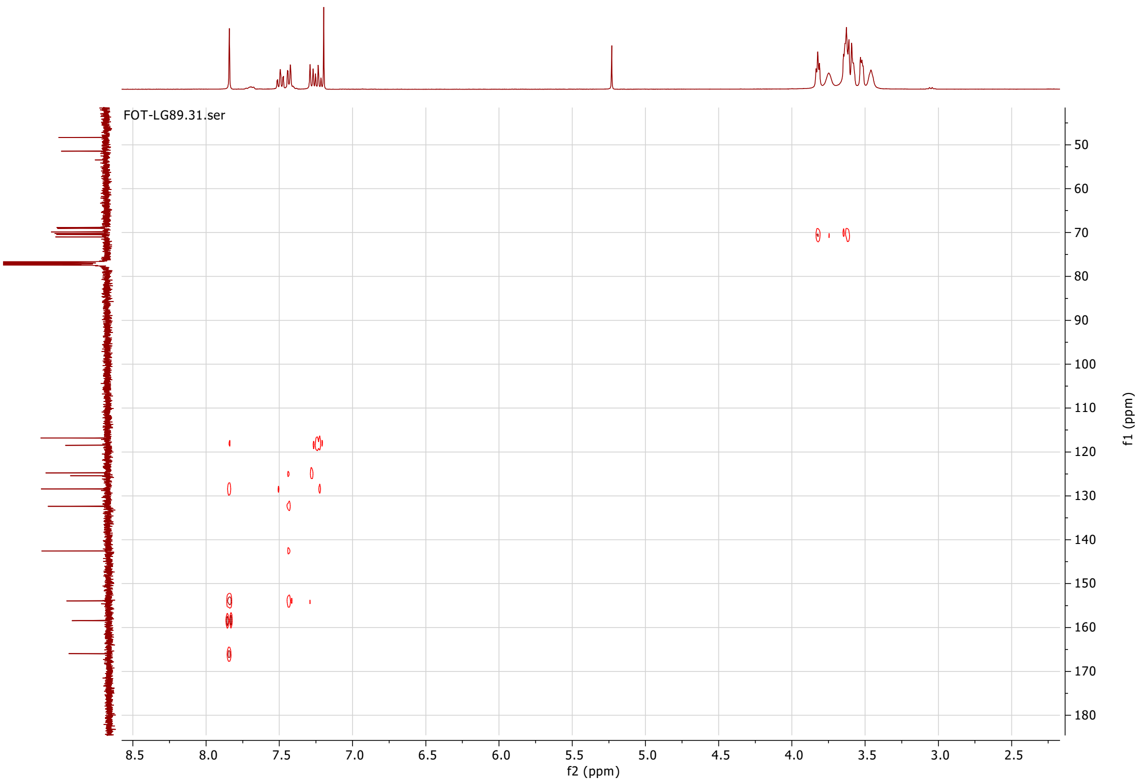

Figure S11. ^{1}H-^{13}C HMBC NMR spectrum of **3b**.

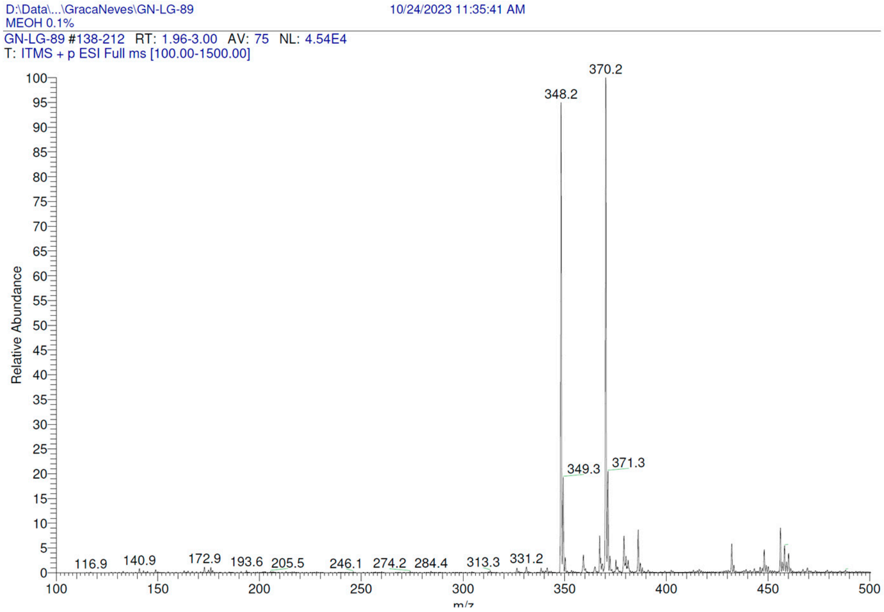

Figure S12. Mass spectrum of **3b**.

Table S3. Full NMR peak assignment for chemosensor **3c**.

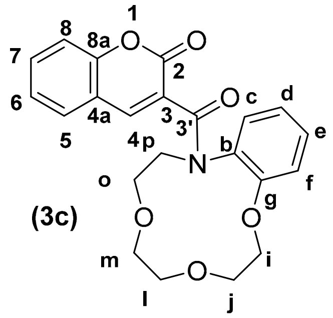

Position	^{1}H	^{13}C
2	-	158.41
3	-	126.13
4	7.69	140.82
4a	-	118.20
5	7.22 – 7.10	128.20
6	7.22 – 7.10	124.55
7	7.45	132.16
8	7.35	116.78
8a	-	153.99
3’	-	165.88
b	-	131.01
c	7.13	129.54
d	6.90	121.31
e	7.30	121.31
f	6.69	112.93
g	-	129.50
i/j/m/l *	3.82 – 3.62/3.95 – 3.89	69.18-71.96
o *	3.98 / 3.49	68.30
p *	4.20 / 3.35	51.11

*diasteriotopic protons

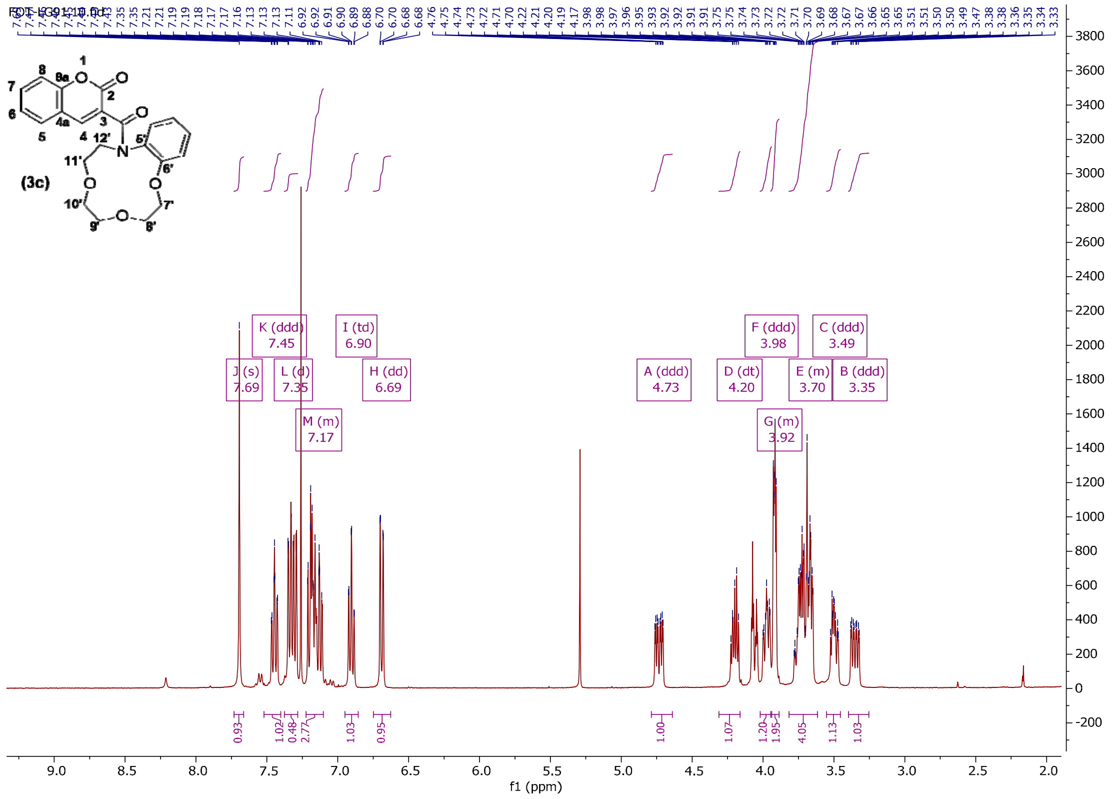

Figure S13. ^{1}H NMR spectrum of **3c**.

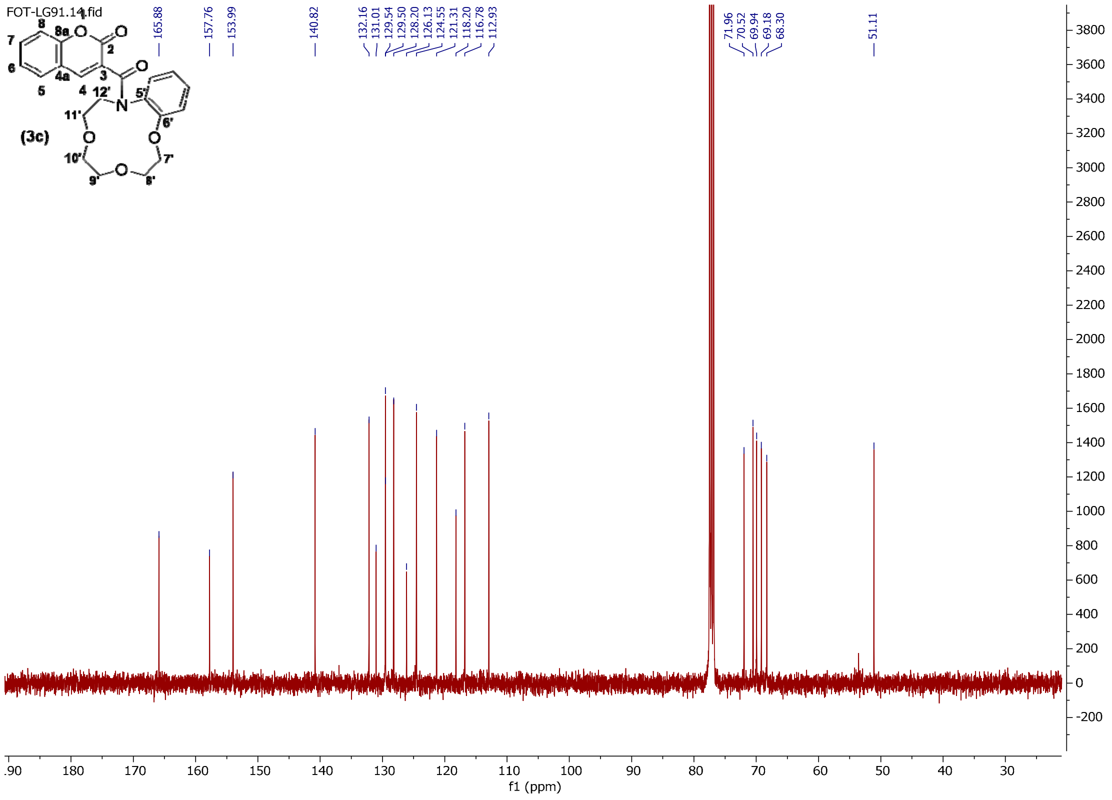

Figure S14. ^{13}C NMR spectrum of **3c**.

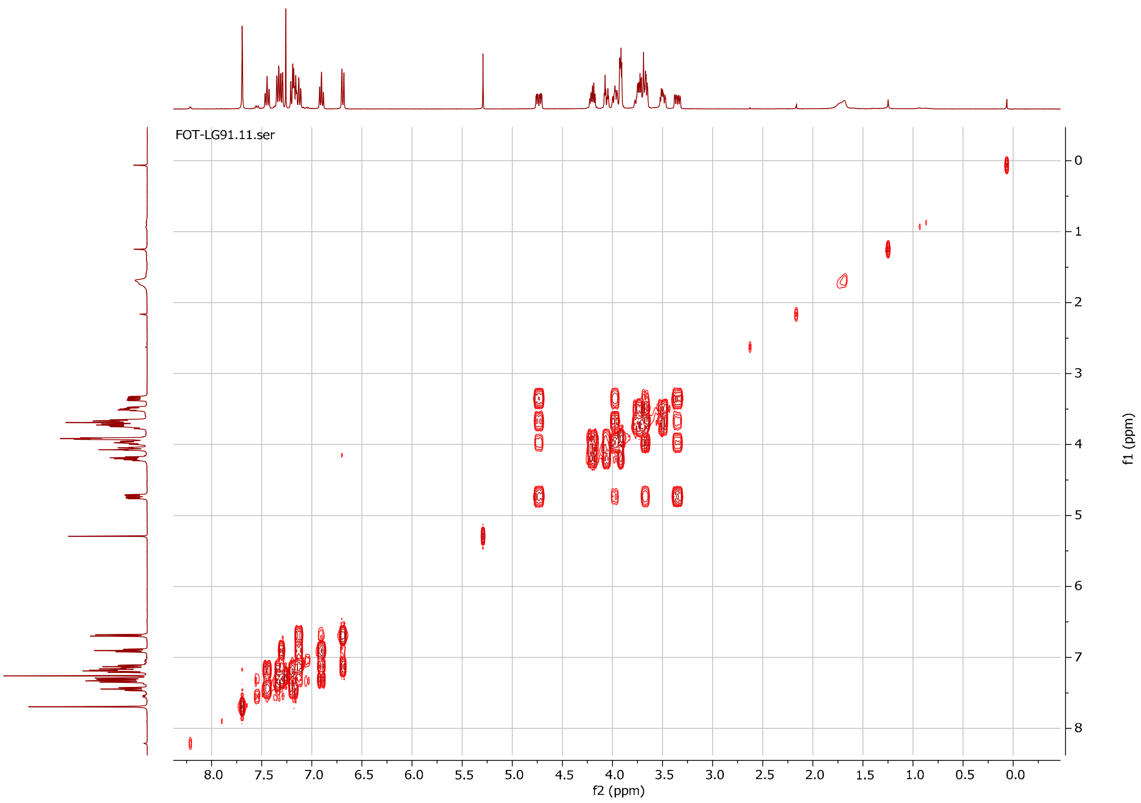

Figure S15. ^{1}H-^{1}H COSY NMR spectrum of **3c**.

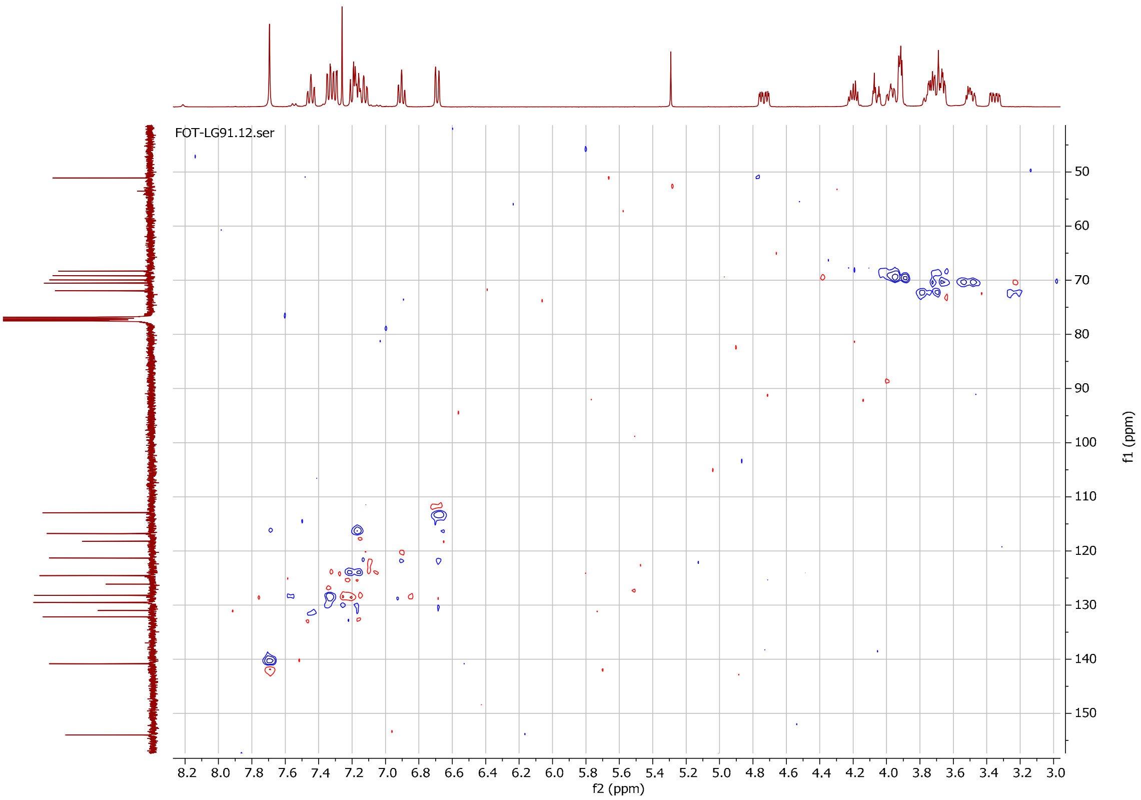

Figure S16. ^{1}H-^{13}C HSQC NMR spectrum of **3c**.

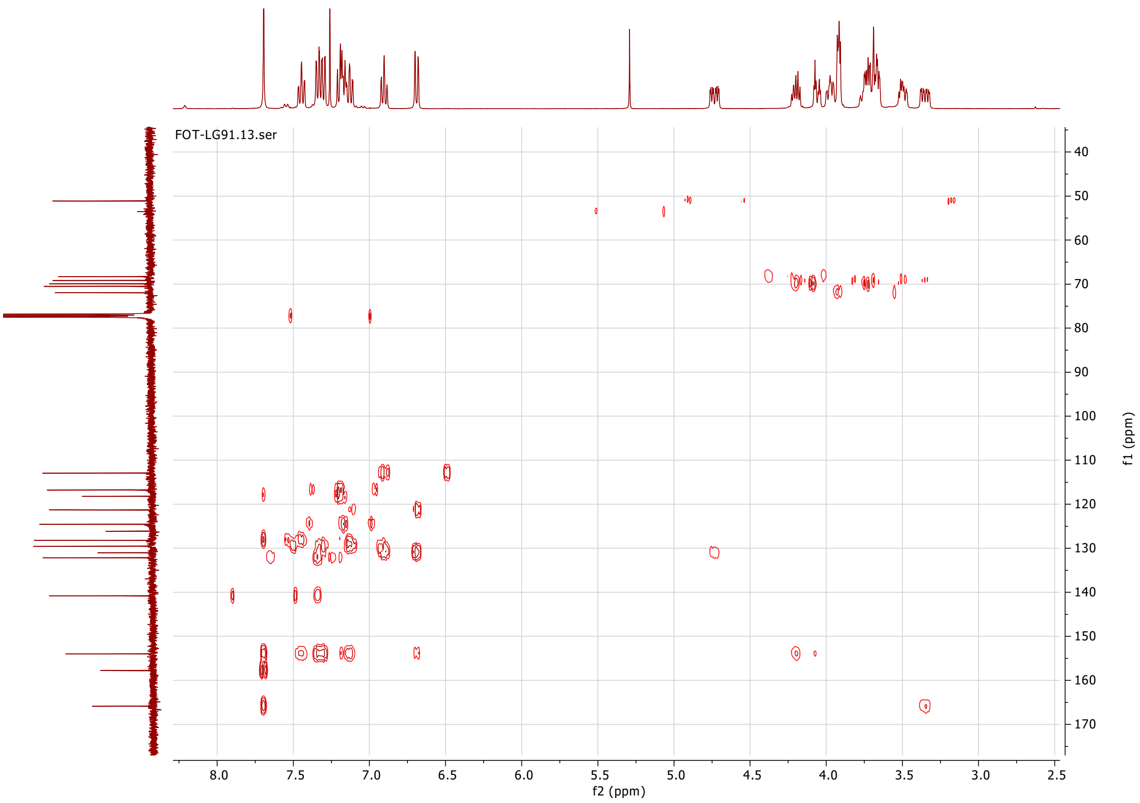

Figure S17. ^{1}H-^{13}C HMBC NMR spectrum of **3c**.

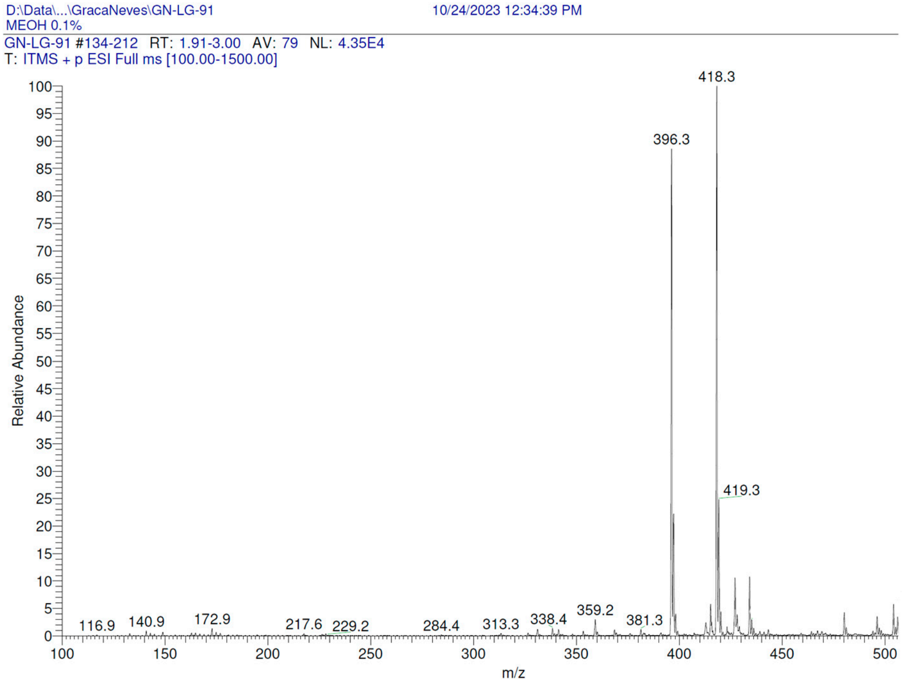

Figure S18. Mass spectrum of **3c**.

Table S4. Full NMR peak assignment for chemosensor **3d**.

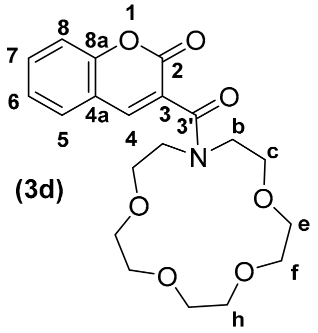

Position	^{1}H	^{13}C
2	-	158.41
3	-	125.75
4	7.88	142.43
4a	-	118.55
5	7.54	128.57
6	7.31	124.94
7	7.54	132.60
8	7.31	116.91
8a	-	154.09
3'	-	165.86
b	3.51	68.88
c/e/f/h	3.82–3.57	48.53-71.48

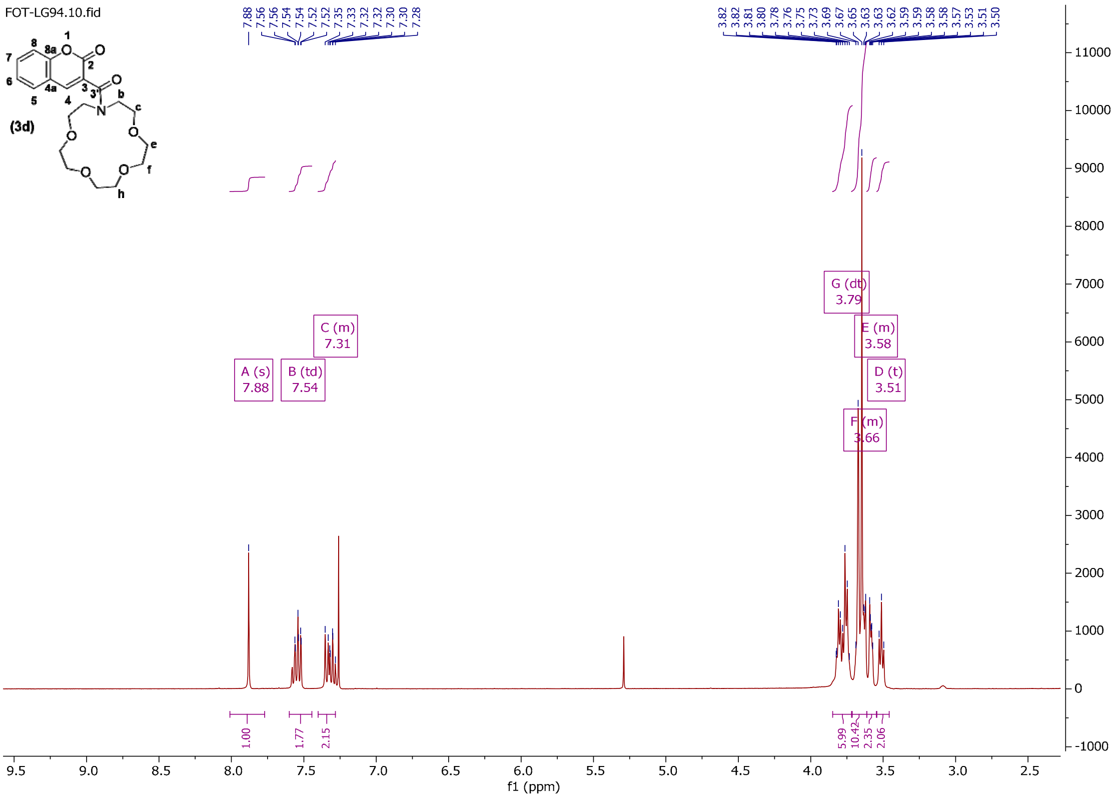

Figure S19. ^{1}H NMR spectrum of **3d**.

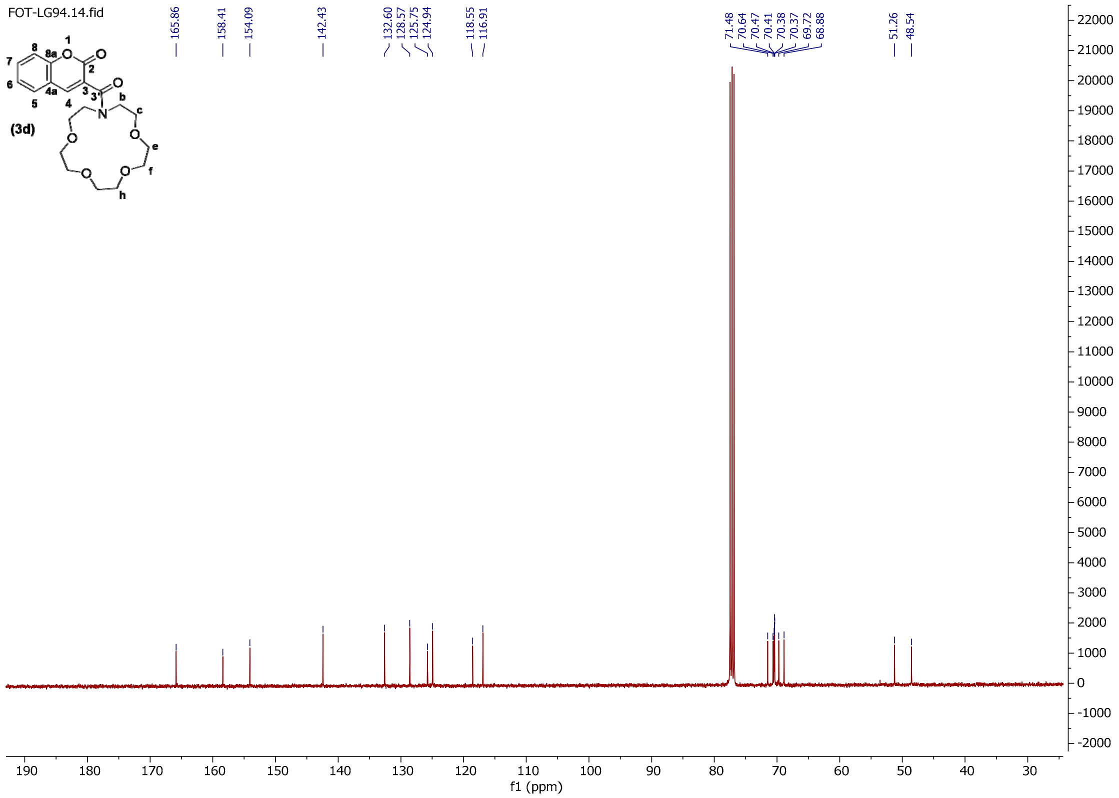

Figure S20. ^{13}C NMR spectrum of **3d**.

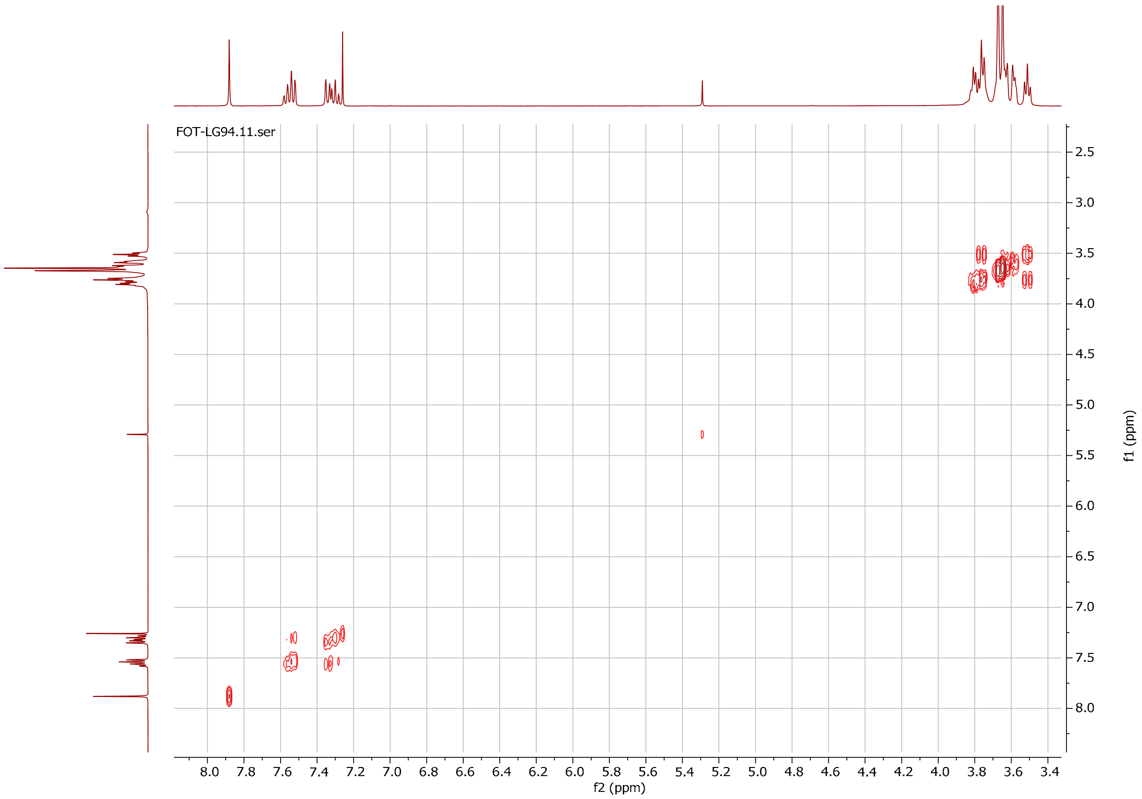

Figure S21. ^{1}H-^{1}H COSY NMR spectrum of **3d**.

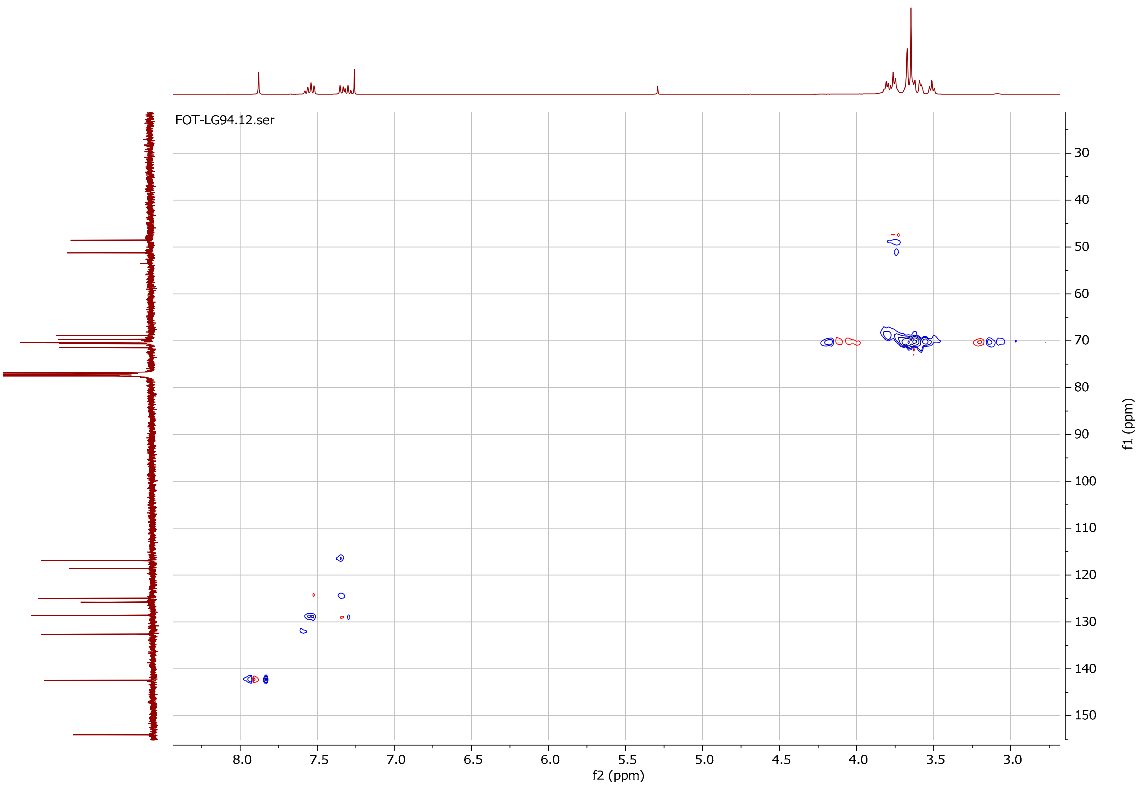

Figure S22. ^{1}H-^{13}C HSQC NMR spectrum of **3d**.

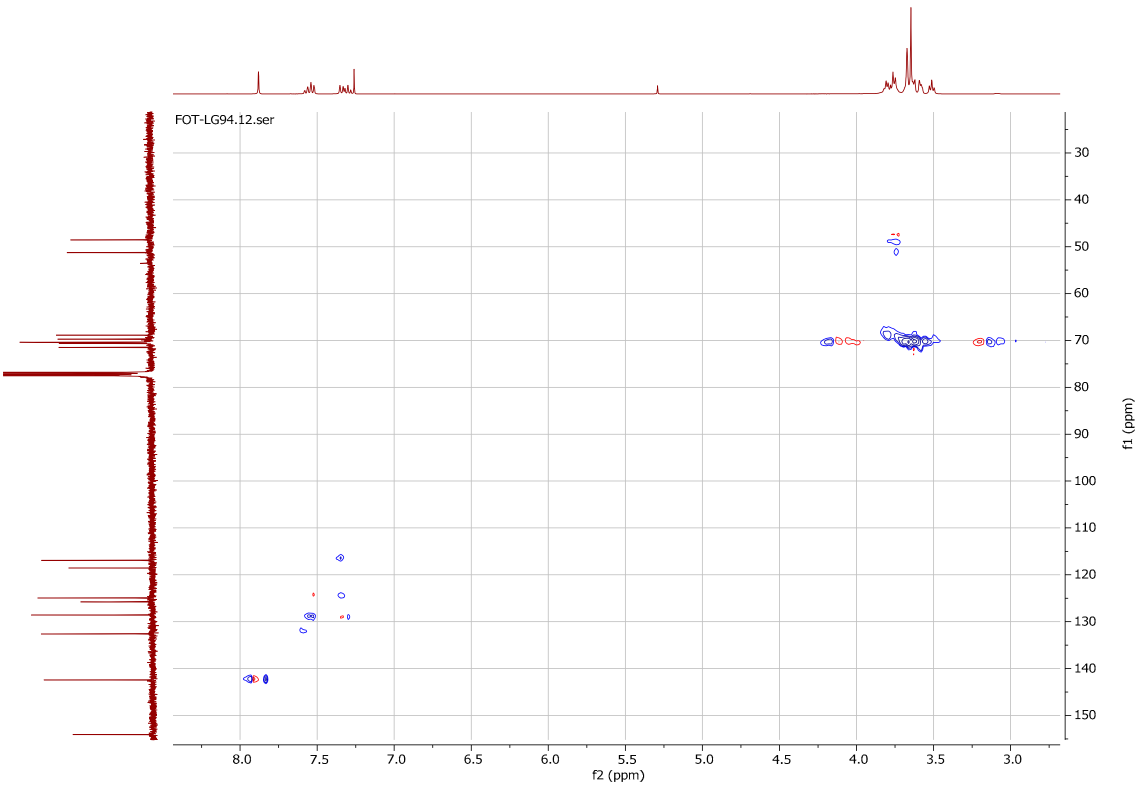

Figure S23. ^{1}H-^{13}C HMBC NMR spectrum of **3d**.

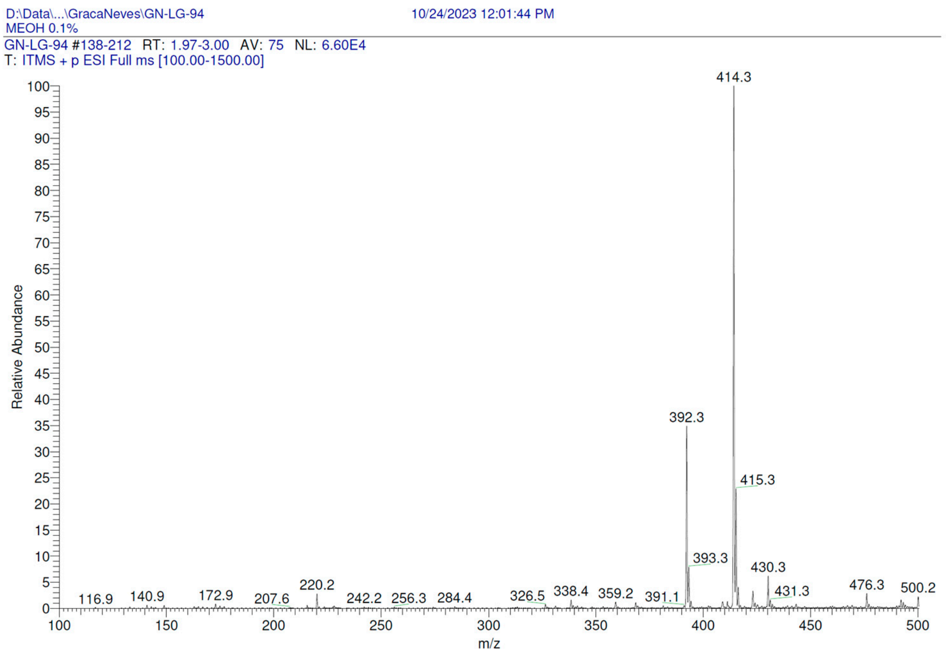

Figure S24. Mass spectrum of **3d**.

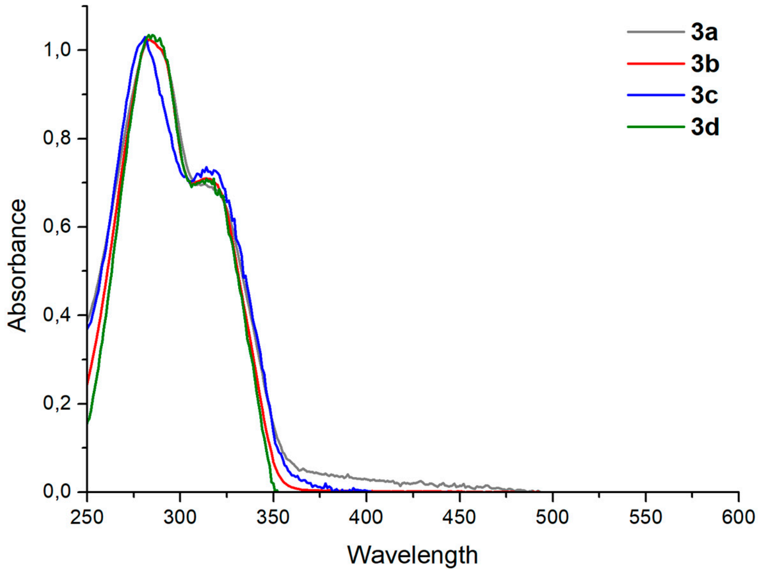

Figure S25. Normalized UV-Vis spectra of compounds **3a-d** in acetonitrile.

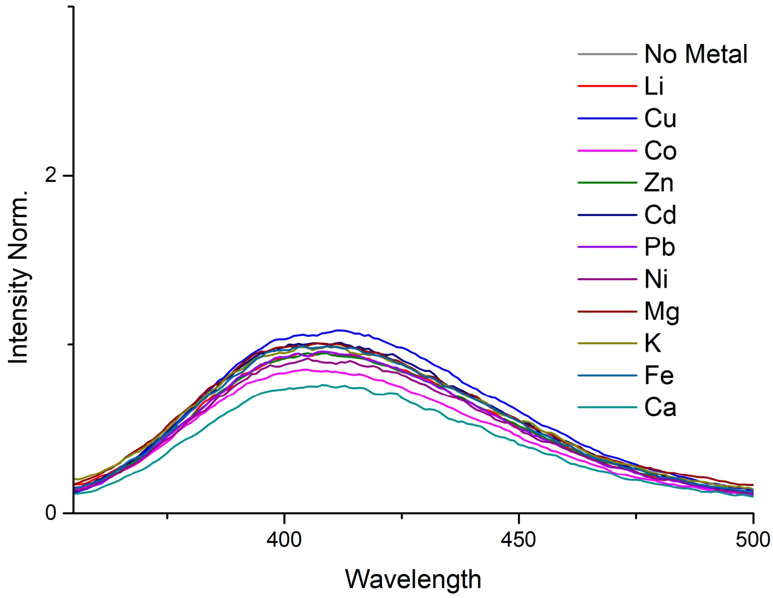

Figure S26. Fluorescent spectra of compounds **3a** against 5 equivelents of several metal cations. For all metals, a spectrum was recorded before and after the addition of 5 eqs. of the corresponding metal, maintaing the concentration of **3a** inalterated. Normalization was performed individually for each metal, being 1 the maximum of the spectra befere the addition of the corresponding metal (λ_{exc} = 310 nm).

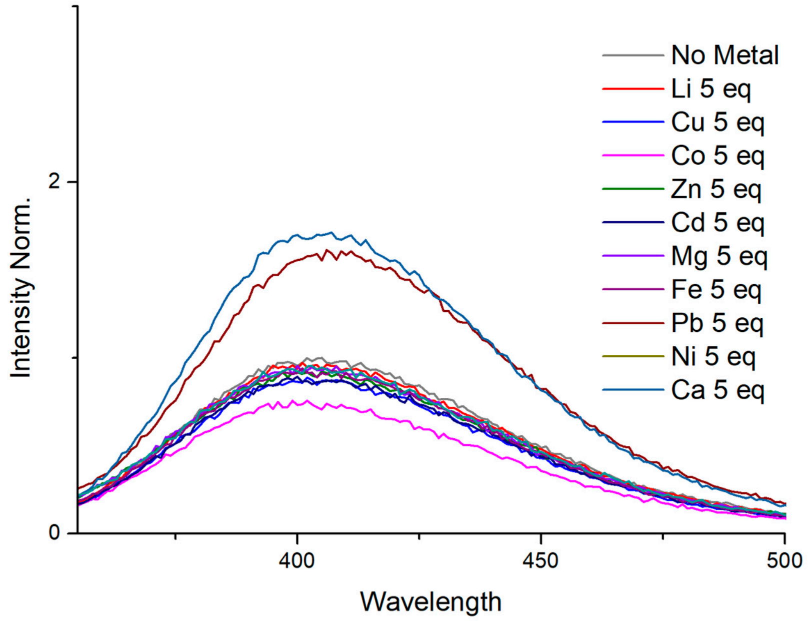

Figure S27. Fluorescent spectra of compounds **3b** against 5 equivelents of several metal cations. For all metals, a spectrum was recorded before and after the addition of 5 eqs. of the corresponding metal, maintaing the concentration of **3b** inalterated. Normalization was performed individually for each metal, being 1 the maximum of the spectra befere the addition of the corresponding metal (λ_{exc} = 315 nm).

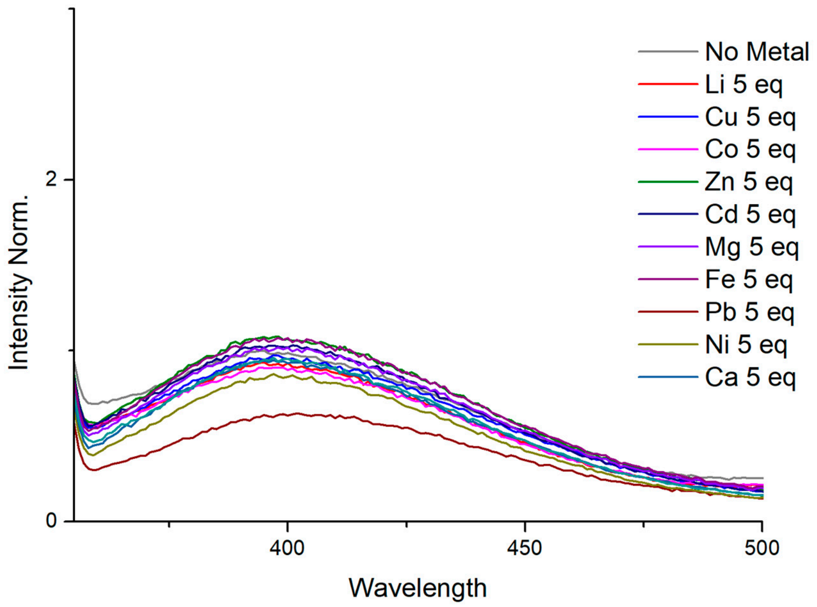

Figure S28. Fluorescent spectra of compounds **3c** against 5 equivelents of several metal cations. For all metals, a spectrum was recorded before and after the addition of 5 eqs. of the corresponding metal, maintaing the concentration of **3c** inalterated. Normalization was performed individually for each metal, being 1 the maximum of the spectra befere the addition of the corresponding metal (λ_{exc} = 320 nm).

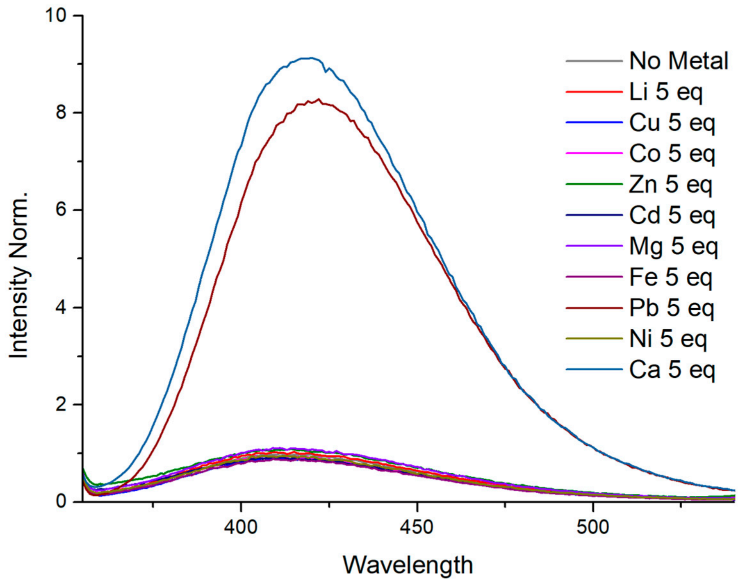

Figure S29. Fluorescent spectra of compounds **3d** against 5 equivelents of several metal cations. For all metals, a spectrum was recorded before and after the addition of 5 eqs. of the corresponding metal, maintaing the concentration of **3d** inalterated. Normalization was performed individually for each metal, being 1 the maximum of the spectra befere the addition of the corresponding metal (λ_{exc} = 315 nm).

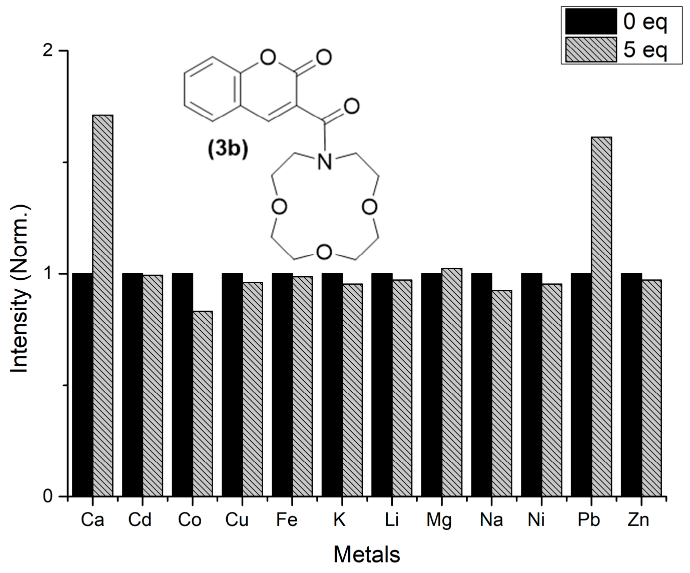

Figure S30. Overall response on the luminescence of compound **3b** against a series of metal cations (λ_{exc} = 315 nm).

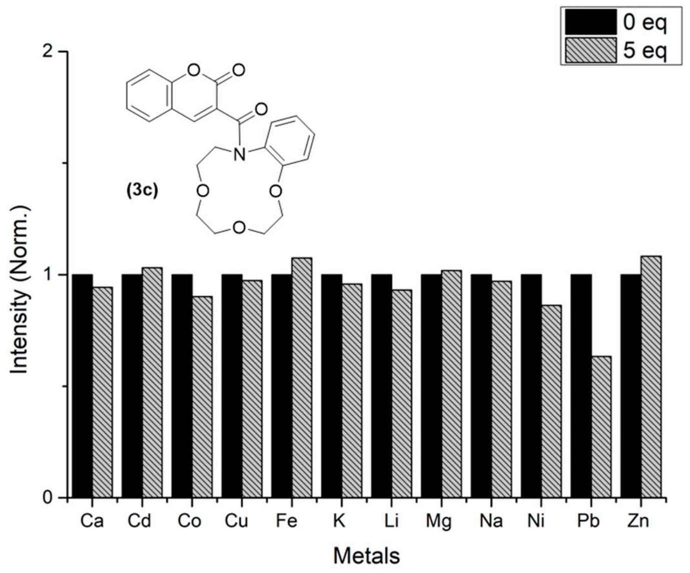

Figure S31. Overall response on the luminescence of compound **3c** against a series of metal cations (λ_{exc} = 320 nm).

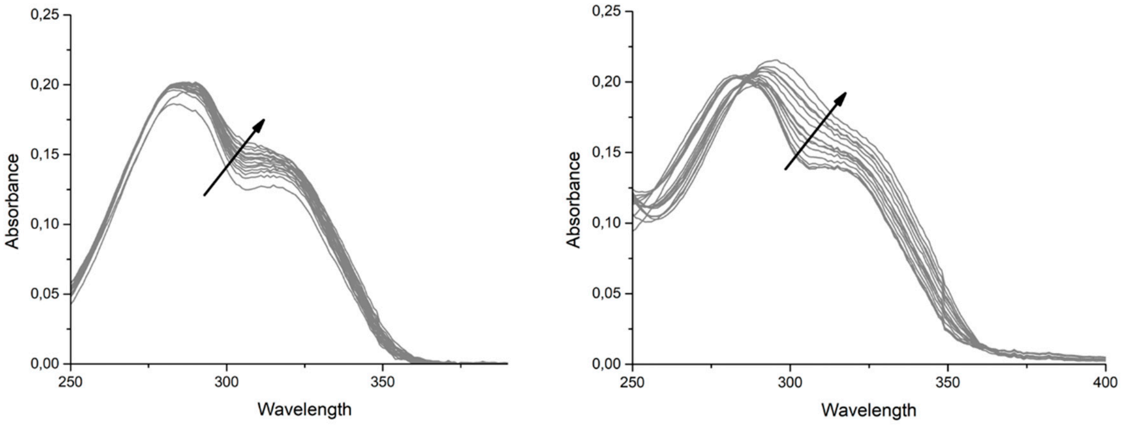

Figure S32. UV-Vis titration of **3d** with Ca^{2+} - **A)** and with Pb^{2+} - **B)**. Conditions: Acetonitrile as solvent.

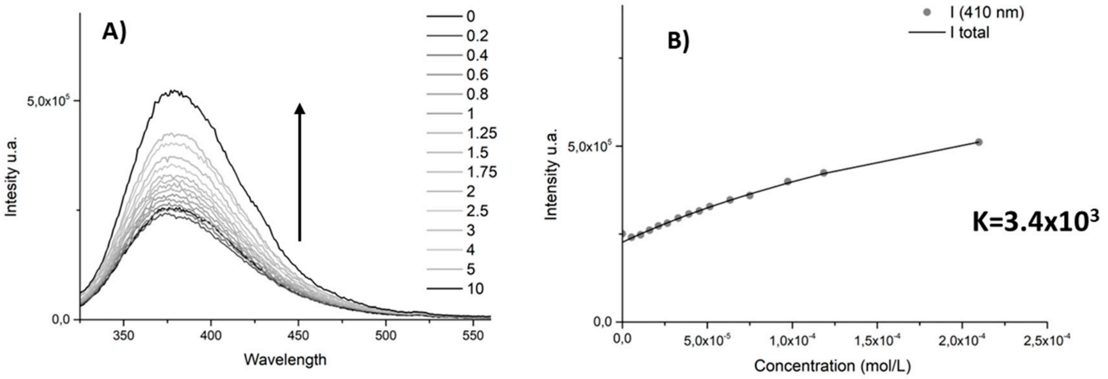

Figure S33. Fluorescence titration (A) and fitting for association constant (B) determination of complexation between compound **3b** and Ca (II). Conditions: variation between 0 and 10 equivalents of lead using acetonitrile as solvent (λ_{exc} = 315 nm).

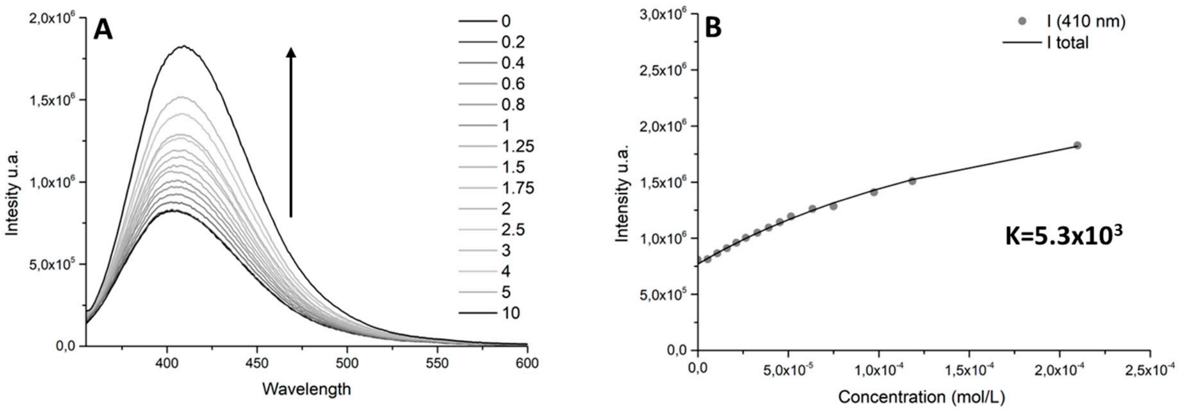

Figure S34. Fluorescence titration (A) and fitting (B) for association constant determination of complexation between compound **3b** and Pb (II). Conditions: variation between 0 and 10 equivalents of lead using acetonitrile as solvent (λ_{exc} = 315 nm).

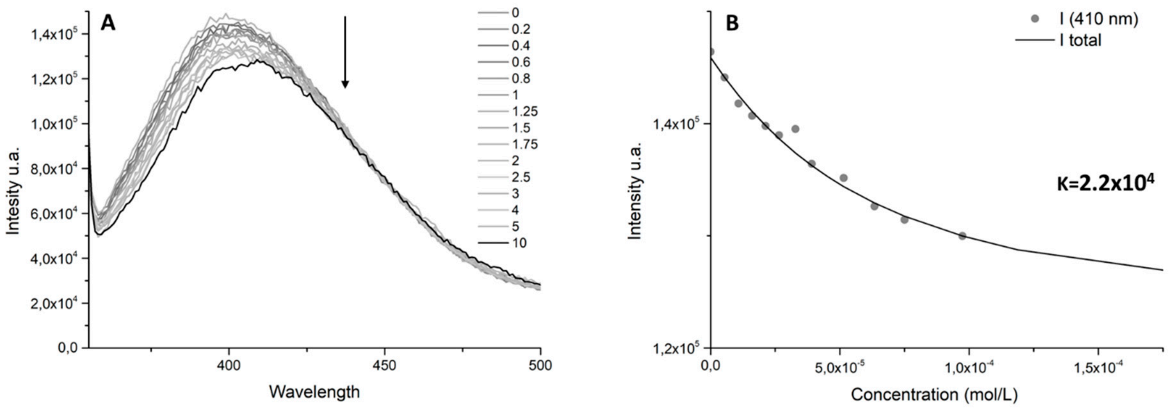

Figure S35. Fluorescence titration (A) and fitting (B) for association constant determination of complexation between compound **3c** and Pb (II). Conditions: variation between 0 and 10 equivalents of lead using acetonitrile as solvent (λ_{exc} = 320 nm).

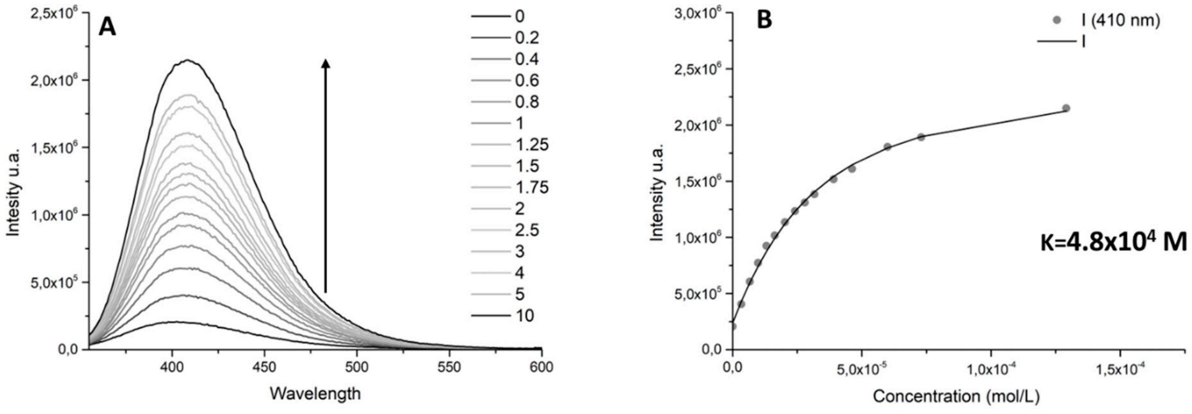

Figure S36. Fluorescence titration (A) and fitting (B) for association constant determination of complexation between compound **3d** and Ca (II). Conditions: variation between 0 and 10 equivalents of lead using acetonitrile as solvent (λ_{exc} = 315 nm).

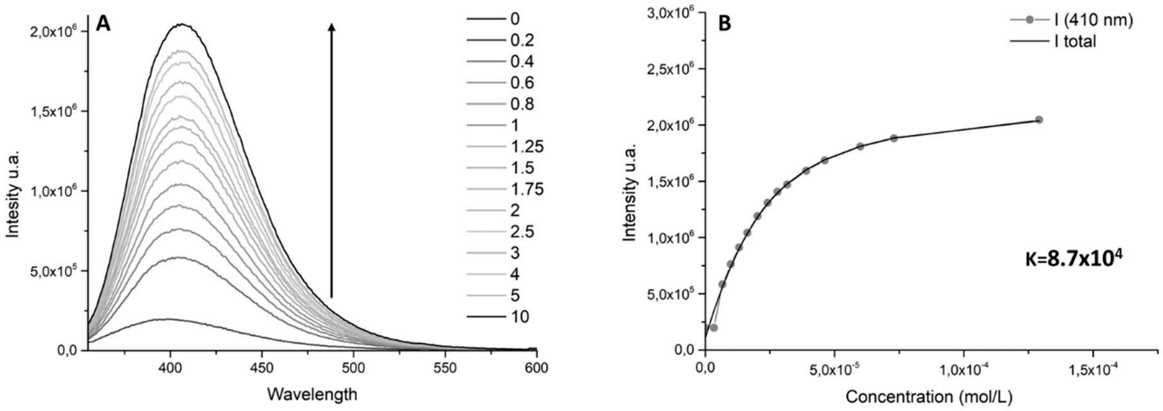

Figure S37. Fluorescence titration (A) and fitting (B) for association constant determination of complexation between compound **3d** and Pb (II). Conditions: variation between 0 and 10 equivalents of lead using acetonitrile as solvent (λ_{exc} = 315 nm).

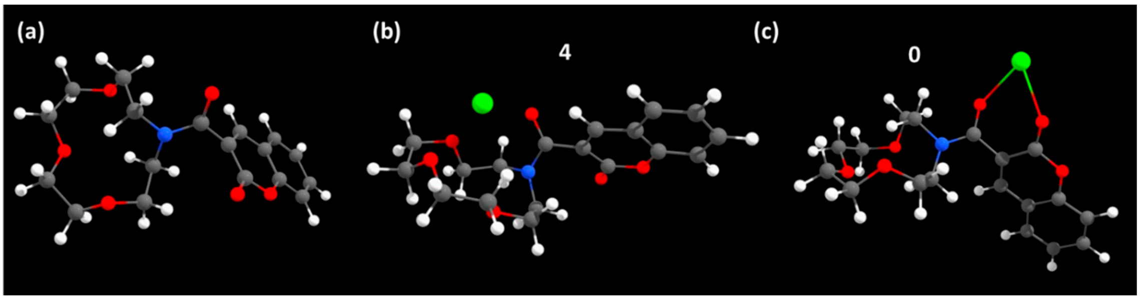

Figure S38. Optimized geometries of **3d** (a), the corresponding Ca^{2+} complex through the azacrown (b) and carbonyl groups (c). Relative energies of (b) and (c) are shown in kcal.mol^{-1}.

Table S5. Electronic configuration of S_1, S_2 and the main involved Molecular Orbitals (MO) in **3d** and **3d-Ca**. f and E are the calculated oscillator strength and excitation energy respectively.

	**State**	**f**	**E (eV)**	**λ (nm)**	**MO (%)**	**Character**
**3d**	S_1	0.0007	3.51	353	H → L (98%)	nπ*
	S_2	0.2711	3.93	315	H -1 → L (86%)	ππ*
					H - 5 → L (9%)	
**3d-Ca**	S_1	0.2312	3.82	325	H → L (88%)	ππ*
					H - 1 → L (9%)	

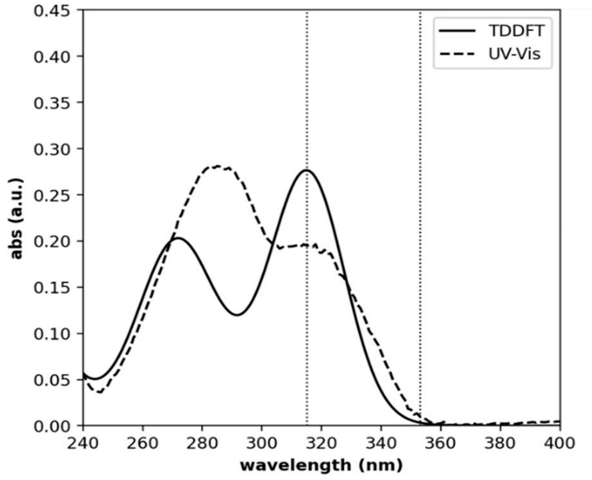

Figure S39. TDDFT simulated spectrum (solid line) overlaid with the experimental UV-Vis of **3d** in MeCN (dashed line). Lowest electronic states S_1 and S_2 are marked by vertical dotted lines.

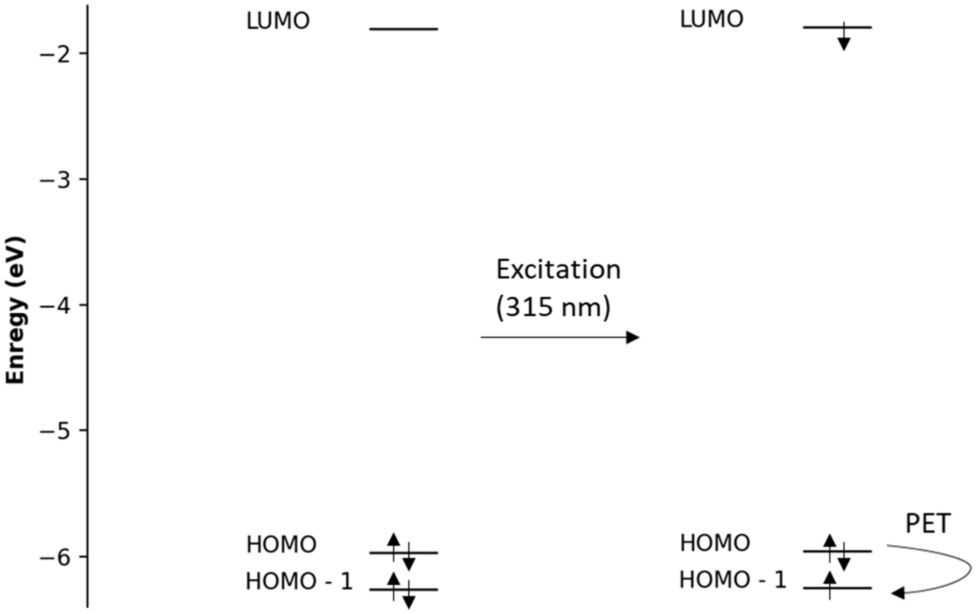

Figure S40. PET process based on frontier MO energy diagram of **3d** upon excitation at 315 nm. (Adapted from reference 21 of the main text).

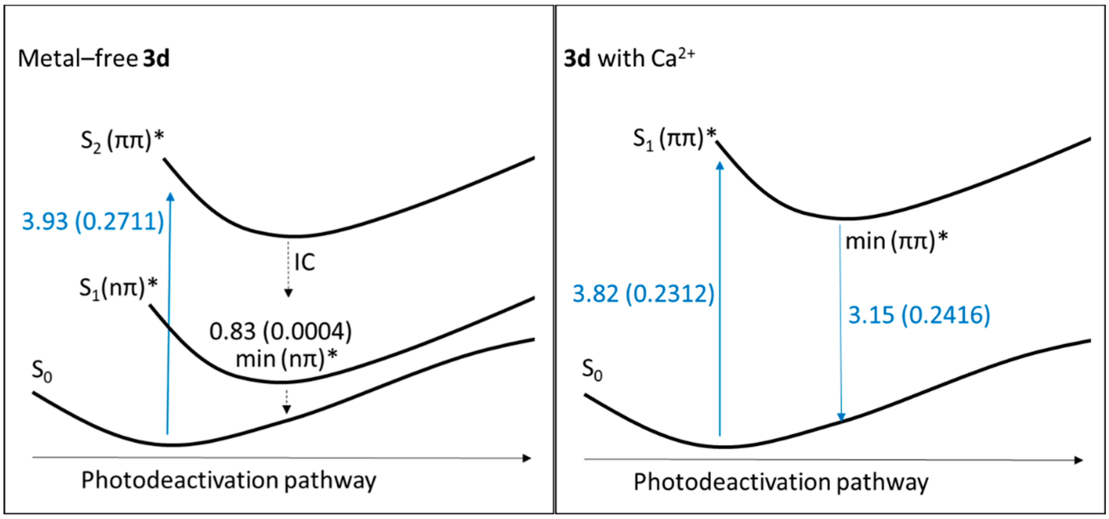

Figure S41. Schematic potential energy surfaces of **3d** without (left) and with Ca^{2+} (right). Radiative transitions are shown with blue arrows and non-radiative ones with dashed black arrows with their respective energies in eV and the oscillator strengths between parentheses. (Adapted from reference 22 of the main text).

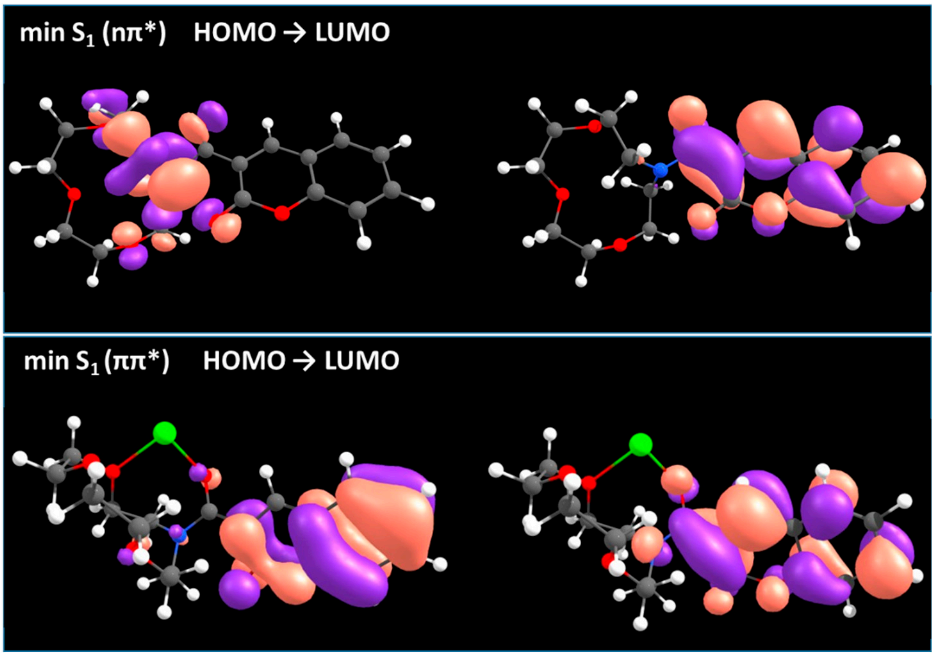

Figure S42. Molecular orbitals involved in the minimum of S_1 state in metal-free **3d** (top) and coordinated to Ca^{2+} (bottom).

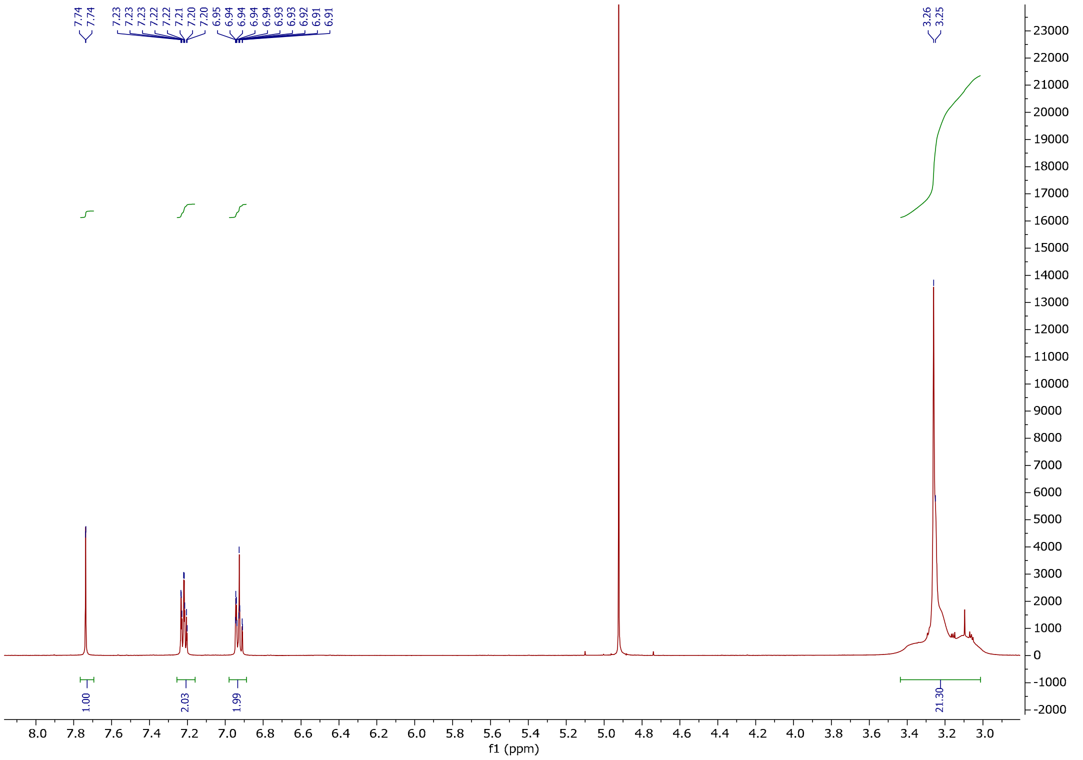

Figure S43. ^{1}H NMR spectrum of **3d.Ca**, solvent acetonitrile.

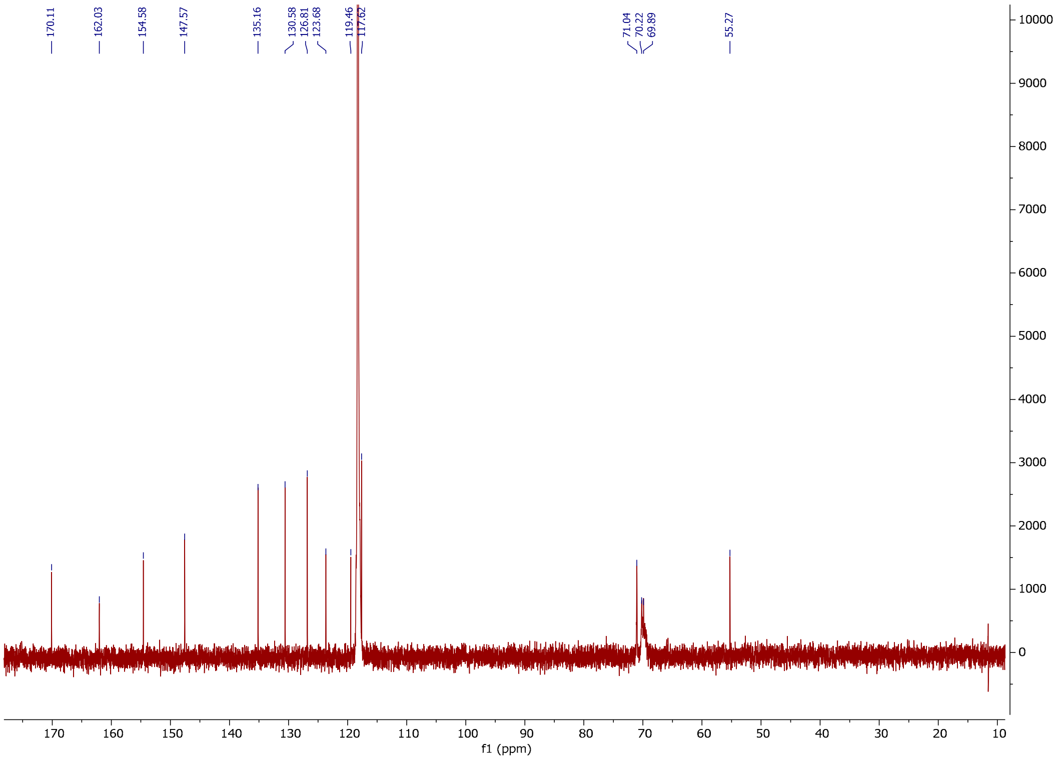

Figure S44. ^{13}C NMR spectrum of **3d.Ca**, deuterated solvent acetonitrile.

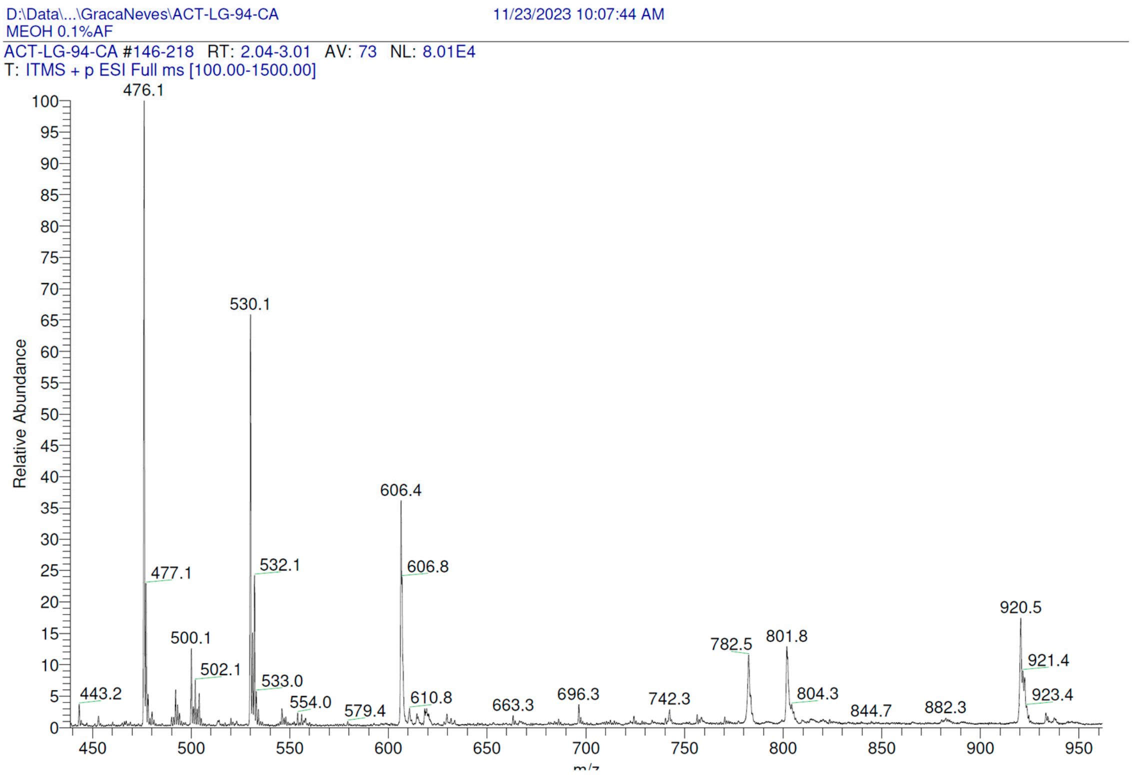

Figure S45. Mass spectrum of **3d.Ca**, molecular ion peak in the presence of two sodium ions.

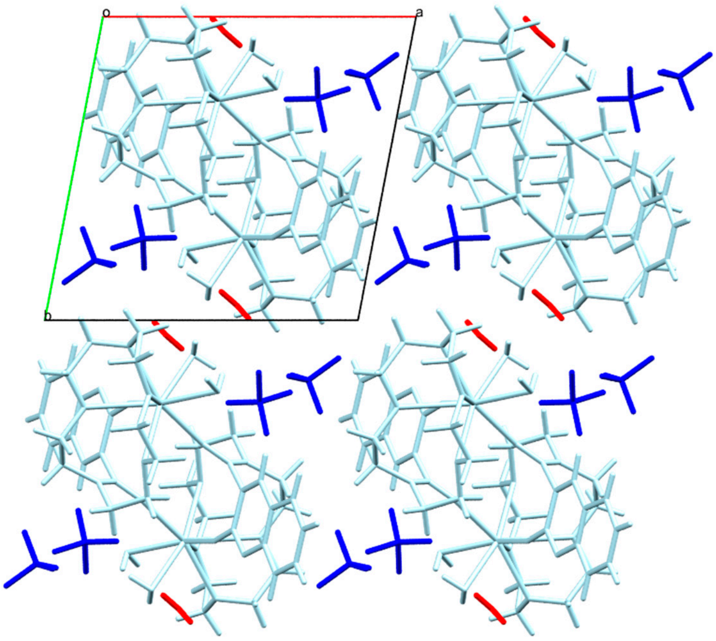

Figure S46. Supramolecular arrangement for complex **3d.Ca**, viewed along the *c* axis. The dication, anion and co-crystallized water molecules are represented in light blue, blue and red, respectively.

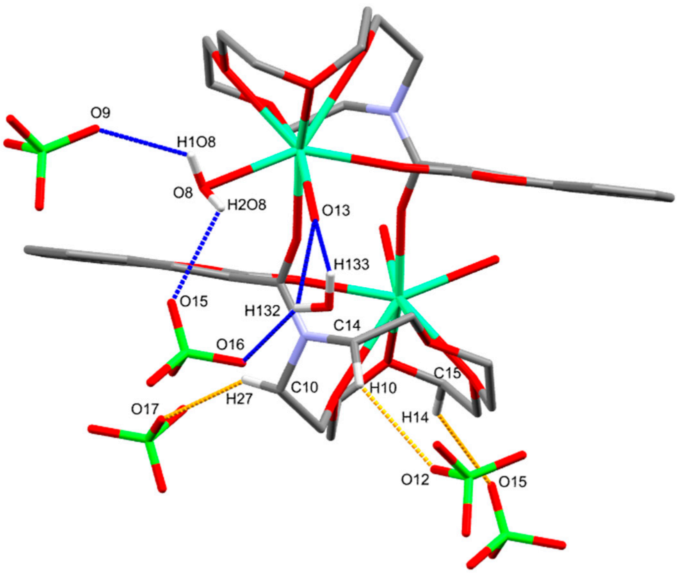

Figure S47. Classical and non-classical hydrogen bonds observed in **3d.Ca**, with O–H…O and C–H…O depicted in blue and orange, respectively.

Table S6. Classical and non-classical hydrogen bonds in the cationic complex **3d.Ca**.

D–H…A	H…A	D…A	D–H…A	Symmetry operation
O8–H108…O9	2.41	2.83(3)	111	
O8–H208…O15	2.44	2.84(4)	108	
O18–H132…O16	2.59	3.32(4)	144	
O18–H133…O13	2.71	2.82(4)	88	
O18–H132…O16	2.55	2.81(3)	99	
C10–H27…O17	2.44	3.28(3)	144	-1+*x*, *y*, *z*
C14–H10…O12	2.57	3.41(2)	146	1-*x*, 1-*y*, -*z*
C15–H14…O15	2.59	3.41(3)	142	1-*x*, -*y*, -*z*

Table S7. Crystal data and structure refinement for compound **3d.Ca**.

Identification code	3d-Ca	
Empirical formula	$C_{40} H_{62} Ca_2 Cl_4 N_2 O_{36}$	
Formula weight	1368.87	
Temperature	293(2) K	
Wavelength	0.71073 Å	
Crystal system	Triclinic	
Space group	P -1	
Unit cell dimensions	a = 11.377(3) Å	α= 111.698(7)°.
	b = 11.924(3) Å	β= 99.104(7)°.
	c = 12.162(3) Å	γ = 96.546(8)°.

Volume	1486.6(6) Å^3
Z	1
Density (calculated)	1.529 Mg/m^3
Absorption coefficient	0.471 mm^{-1}
F(000)	712
Crystal size	0.200 x 0.120 x 0.080 mm^3
Theta range for data collection	1.846 to 25.652°.
Index ranges	-13<=h<=13, -14<=k<=14, -14<=l<=14
Reflections collected	51514
Independent reflections	5609 [R(int) = 0.3228]
Completeness to theta = 25.242°	99.9 %
Absorption correction	Semi-empirical from equivalents
Max. and min. transmission	0.7453 and 0.4132
Refinement method	Full-matrix least-squares on F^2
Data / restraints / parameters	5609 / 0 / 373
Goodness-of-fit on F^2	1.023
Final R indices [I>2sigma(I)]	R1 = 0.1417, wR2 = 0.3158
R indices (all data)	R1 = 0.2582, wR2 = 0.3550
Extinction coefficient	n/a
Largest diff. peak and hole	1.291 and -0.619 e.Å^{-3}

Table S8. Atomic coordinates (x 10^4) and equivalent isotropic displacement parameters ($Å^2$x 10^3)
for 3d-ca. U(eq) is defined as one third of the trace of the orthogonalized U^{ij} tensor.

	x	y	z	U(eq)
C(1)	457(14)	3120(30)	8174(19)	117(8)
C(2)	981(12)	4095(17)	7922(13)	90(5)
C(3)	1589(9)	3814(13)	6968(10)	51(3)
C(4)	2126(9)	4707(11)	6593(9)	46(3)
C(5)	2627(8)	4399(9)	5617(8)	38(2)
C(6)	3233(9)	5304(9)	5188(8)	37(2)
C(7)	3170(15)	1321(12)	-490(10)	87(5)
C(8)	1600(11)	707(11)	1298(13)	77(4)
C(9)	1036(10)	1756(10)	1235(12)	72(4)
C(10)	1447(8)	4731(10)	3530(9)	50(3)
C(11)	3657(11)	3506(11)	139(10)	59(3)
C(12)	4420(11)	4604(10)	1145(9)	54(3)
C(13)	4359(10)	5849(9)	3209(10)	52(3)
C(14)	3271(10)	6209(9)	3716(9)	46(3)
C(15)	3130(14)	416(12)	54(12)	78(4)
C(16)	1252(10)	3896(10)	2185(10)	54(3)
C(17)	2699(8)	3097(9)	4987(9)	40(2)
C(18)	1622(9)	2610(14)	6319(11)	61(3)
C(19)	1082(12)	1658(15)	6566(14)	86(4)
C(20)	500(16)	1990(20)	7530(20)	110(7)
N(1)	2700(7)	5334(7)	4147(6)	38(2)
O(1)	4213(6)	5935(6)	5785(6)	47(2)
O(2)	3790(7)	2468(7)	429(6)	53(2)
O(3)	1803(6)	2863(6)	2080(6)	55(2)
O(4)	4040(6)	4666(6)	2248(6)	45(2)
O(5)	2846(7)	832(6)	1213(7)	55(2)
O(6)	3218(7)	2736(6)	4163(6)	51(2)
O(7)	2195(7)	2273(7)	5374(6)	56(2)
O(8)	5952	2334	1726	70(2)
O(9)	6364(17)	2720(30)	-350(20)	292(15)
O(10)	8266(10)	2377(13)	-126(14)	141(5)
O(11)	7150(30)	1720(20)	-1920(20)	277(13)

O(12)	7768(13)	3728(13)	-953(13)	141(5)
O(13)	4957	1126	3190	116(4)
O(14)	9610(20)	1250(20)	3350(20)	273(14)
O(15)	8140(30)	1840(40)	2730(20)	510(40)
O(16)	8230(30)	1810(30)	4450(40)	360(20)
O(17)	9350(30)	3010(15)	4080(30)	335(18)
O(18)	6039	-492	4038	340(20)
Cl(1)	7363(4)	2693(4)	-750(4)	87(1)
Cl(2)	8776(3)	1973(3)	3632(3)	61(1)
Ca(1)	4193(2)	2729(2)	2578(2)	37(1)

Table S9. Bond lengths [Å] and angles [°] for 3d-ca.

C(1)-C(20)	1.29(3)
C(1)-C(2)	1.40(2)
C(1)-H(1)	0.9300
C(2)-C(3)	1.398(17)
C(2)-H(21)	0.9300
C(3)-C(18)	1.367(17)
C(3)-C(4)	1.420(15)
C(4)-C(5)	1.344(13)
C(4)-H(22)	0.9300
C(5)-C(17)	1.474(14)
C(5)-C(6)	1.507(13)
C(6)-O(1)	1.218(11)
C(6)-N(1)	1.330(11)
C(7)-O(2)	1.421(13)
C(7)-C(15)	1.459(18)
C(7)-H(2)	0.9700
C(7)-H(13)	0.9700
C(8)-O(5)	1.433(13)
C(8)-C(9)	1.489(17)
C(8)-H(3)	0.9700
C(8)-H(28)	0.9700
C(9)-O(3)	1.418(12)
C(9)-H(18)	0.9700
C(9)-H(19)	0.9700
C(10)-N(1)	1.462(12)
C(10)-C(16)	1.532(15)
C(10)-H(4)	0.9700
C(10)-H(27)	0.9700
C(11)-O(2)	1.424(13)
C(11)-C(12)	1.478(15)
C(11)-H(5)	0.9700
C(11)-H(12)	0.9700
C(12)-O(4)	1.453(12)
C(12)-H(6)	0.9700
C(12)-H(7)	0.9700
C(13)-O(4)	1.421(12)

C(13)-C(14)	1.504(15)
C(13)-H(8)	0.9700
C(13)-H(11)	0.9700
C(14)-N(1)	1.464(12)
C(14)-H(9)	0.9700
C(14)-H(10)	0.9700
C(15)-O(5)	1.414(14)
C(15)-Ca(1)	3.209(13)
C(15)-H(14)	0.9700
C(15)-H(15)	0.9700
C(16)-O(3)	1.418(12)
C(16)-H(16)	0.9700
C(16)-H(17)	0.9700
C(17)-O(6)	1.210(11)
C(17)-O(7)	1.347(11)
C(18)-O(7)	1.365(14)
C(18)-C(19)	1.381(17)
C(19)-C(20)	1.39(2)
C(19)-H(20)	0.9300
C(20)-H(23)	0.9300
O(1)-Ca(1)#1	2.376(7)
O(2)-Ca(1)	2.475(7)
O(3)-Ca(1)	2.727(7)
O(4)-Ca(1)	2.505(6)
O(5)-Ca(1)	2.418(7)
O(6)-Ca(1)	2.369(7)
O(8)-Ca(1)	2.410(2)
O(8)-H(1O8)	0.84950
O(8)-H(2O8)	0.84921
O(9)-Cl(1)	1.306(13)
O(10)-Cl(1)	1.351(11)
O(11)-Cl(1)	1.43(2)
O(12)-Cl(1)	1.389(12)
O(13)-Ca(1)	2.488(2)
O(13)-H(173)	0.84992
O(13)-H(273)	0.84999
O(14)-Cl(2)	1.350(16)
O(15)-Cl(2)	1.159(17)

O(16)-Cl(2)	1.30(2)
O(17)-Cl(2)	1.205(16)
O(18)-H(132)	0.85115
O(18)-H(133)	0.83363
Ca(1)-H(1O8)	2.619
Ca(1)-H(2O8)	2.500
Ca(1)-H(173)	2.570
Ca(1)-H(273)	2.705

C(20)-C(1)-C(2)	121.6(18)
C(20)-C(1)-H(1)	119.2
C(2)-C(1)-H(1)	119.2
C(3)-C(2)-C(1)	117.7(17)
C(3)-C(2)-H(21)	121.2
C(1)-C(2)-H(21)	121.2
C(18)-C(3)-C(2)	118.7(12)
C(18)-C(3)-C(4)	117.7(10)
C(2)-C(3)-C(4)	123.5(13)
C(5)-C(4)-C(3)	122.2(11)
C(5)-C(4)-H(22)	118.9
C(3)-C(4)-H(22)	118.9
C(4)-C(5)-C(17)	118.0(9)
C(4)-C(5)-C(6)	124.7(9)
C(17)-C(5)-C(6)	117.0(8)
O(1)-C(6)-N(1)	123.1(9)
O(1)-C(6)-C(5)	118.4(8)
N(1)-C(6)-C(5)	118.4(8)
O(2)-C(7)-C(15)	107.7(10)
O(2)-C(7)-H(2)	110.2
C(15)-C(7)-H(2)	110.2
O(2)-C(7)-H(13)	110.2
C(15)-C(7)-H(13)	110.2
H(2)-C(7)-H(13)	108.5
O(5)-C(8)-C(9)	113.4(10)
O(5)-C(8)-H(3)	108.9
C(9)-C(8)-H(3)	108.9
O(5)-C(8)-H(28)	108.9
C(9)-C(8)-H(28)	108.9

H(3)-C(8)-H(28)	107.7
O(3)-C(9)-C(8)	108.3(9)
O(3)-C(9)-H(18)	110.0
C(8)-C(9)-H(18)	110.0
O(3)-C(9)-H(19)	110.0
C(8)-C(9)-H(19)	110.0
H(18)-C(9)-H(19)	108.4
N(1)-C(10)-C(16)	115.8(9)
N(1)-C(10)-H(4)	108.3
C(16)-C(10)-H(4)	108.3
N(1)-C(10)-H(27)	108.3
C(16)-C(10)-H(27)	108.3
H(4)-C(10)-H(27)	107.4
O(2)-C(11)-C(12)	107.9(9)
O(2)-C(11)-H(5)	110.1
C(12)-C(11)-H(5)	110.1
O(2)-C(11)-H(12)	110.1
C(12)-C(11)-H(12)	110.1
H(5)-C(11)-H(12)	108.4
O(4)-C(12)-C(11)	107.3(8)
O(4)-C(12)-H(6)	110.3
C(11)-C(12)-H(6)	110.3
O(4)-C(12)-H(7)	110.3
C(11)-C(12)-H(7)	110.3
H(6)-C(12)-H(7)	108.5
O(4)-C(13)-C(14)	110.4(8)
O(4)-C(13)-H(8)	109.6
C(14)-C(13)-H(8)	109.6
O(4)-C(13)-H(11)	109.6
C(14)-C(13)-H(11)	109.6
H(8)-C(13)-H(11)	108.1
N(1)-C(14)-C(13)	113.7(8)
N(1)-C(14)-H(9)	108.8
C(13)-C(14)-H(9)	108.8
N(1)-C(14)-H(10)	108.8
C(13)-C(14)-H(10)	108.8
H(9)-C(14)-H(10)	107.7
O(5)-C(15)-C(7)	114.7(11)

O(5)-C(15)-Ca(1)	44.7(5)
C(7)-C(15)-Ca(1)	85.2(7)
O(5)-C(15)-H(14)	108.6
C(7)-C(15)-H(14)	108.6
Ca(1)-C(15)-H(14)	153.0
O(5)-C(15)-H(15)	108.6
C(7)-C(15)-H(15)	108.6
Ca(1)-C(15)-H(15)	88.7
H(14)-C(15)-H(15)	107.6
O(3)-C(16)-C(10)	108.8(8)
O(3)-C(16)-H(16)	109.9
C(10)-C(16)-H(16)	109.9
O(3)-C(16)-H(17)	109.9
C(10)-C(16)-H(17)	109.9
H(16)-C(16)-H(17)	108.3
O(6)-C(17)-O(7)	118.3(10)
O(6)-C(17)-C(5)	123.3(8)
O(7)-C(17)-C(5)	118.3(9)
O(7)-C(18)-C(3)	121.6(10)
O(7)-C(18)-C(19)	115.6(14)
C(3)-C(18)-C(19)	122.8(13)
C(18)-C(19)-C(20)	116.0(16)
C(18)-C(19)-H(20)	122.0
C(20)-C(19)-H(20)	122.0
C(1)-C(20)-C(19)	123.2(18)
C(1)-C(20)-H(23)	118.4
C(19)-C(20)-H(23)	118.4
C(6)-N(1)-C(10)	123.0(8)
C(6)-N(1)-C(14)	119.2(8)
C(10)-N(1)-C(14)	116.4(8)
C(6)-O(1)-Ca(1)#1	162.9(6)
C(7)-O(2)-C(11)	114.8(9)
C(7)-O(2)-Ca(1)	119.9(7)
C(11)-O(2)-Ca(1)	119.4(6)
C(9)-O(3)-C(16)	111.5(8)
C(9)-O(3)-Ca(1)	114.9(6)
C(16)-O(3)-Ca(1)	129.9(6)
C(13)-O(4)-C(12)	114.4(7)

C(13)-O(4)-Ca(1)	122.7(6)
C(12)-O(4)-Ca(1)	111.0(5)
C(15)-O(5)-C(8)	119.1(10)
C(15)-O(5)-Ca(1)	111.0(7)
C(8)-O(5)-Ca(1)	117.4(6)
C(17)-O(6)-Ca(1)	159.8(7)
C(17)-O(7)-C(18)	122.0(9)
Ca(1)-O(8)-H(1O8)	94.62
Ca(1)-O(8)-H(2O8)	86.03
H(1O8)-O(8)-H(2O8)	107.808
Ca(1)-O(13)-H(173)	85.87
Ca(1)-O(13)-H(273)	95.49
H(173)-O(13)-H(273)	107.702
H(132)-O(18)-H(133)	109.132
O(9)-Cl(1)-O(10)	114.9(10)
O(9)-Cl(1)-O(12)	114.5(12)
O(10)-Cl(1)-O(12)	111.9(8)
O(9)-Cl(1)-O(11)	109.5(18)
O(10)-Cl(1)-O(11)	101.2(14)
O(12)-Cl(1)-O(11)	103.3(11)
O(15)-Cl(2)-O(17)	108(3)
O(15)-Cl(2)-O(16)	115(3)
O(17)-Cl(2)-O(16)	107.1(19)
O(15)-Cl(2)-O(14)	107.8(16)
O(17)-Cl(2)-O(14)	105(2)
O(16)-Cl(2)-O(14)	113.0(16)
O(6)-Ca(1)-O(1)#1	82.9(2)
O(6)-Ca(1)-O(8)	146.01(19)
O(1)#1-Ca(1)-O(8)	78.15(18)
O(6)-Ca(1)-O(5)	90.1(3)
O(1)#1-Ca(1)-O(5)	158.3(3)
O(8)-Ca(1)-O(5)	97.15(19)
O(6)-Ca(1)-O(2)	142.6(3)
O(1)#1-Ca(1)-O(2)	129.2(2)
O(8)-Ca(1)-O(2)	68.75(18)
O(5)-Ca(1)-O(2)	65.9(3)
O(6)-Ca(1)-O(13)	75.88(18)
O(1)#1-Ca(1)-O(13)	82.87(18)

O(8)-Ca(1)-O(13)	73.97(6)
O(5)-Ca(1)-O(13)	75.52(18)
O(2)-Ca(1)-O(13)	120.93(19)
O(6)-Ca(1)-O(4)	109.0(2)
O(1)#1-Ca(1)-O(4)	82.0(2)
O(8)-Ca(1)-O(4)	96.11(17)
O(5)-Ca(1)-O(4)	119.6(2)
O(2)-Ca(1)-O(4)	65.1(2)
O(13)-Ca(1)-O(4)	163.39(18)
O(6)-Ca(1)-O(3)	67.4(2)
O(1)#1-Ca(1)-O(3)	129.9(2)
O(8)-Ca(1)-O(3)	144.77(17)
O(5)-Ca(1)-O(3)	64.2(2)
O(2)-Ca(1)-O(3)	76.2(2)
O(13)-Ca(1)-O(3)	124.04(17)
O(4)-Ca(1)-O(3)	71.6(2)
O(6)-Ca(1)-C(15)	114.4(3)
O(1)#1-Ca(1)-C(15)	153.2(3)
O(8)-Ca(1)-C(15)	76.5(3)
O(5)-Ca(1)-C(15)	24.3(3)
O(2)-Ca(1)-C(15)	46.1(3)
O(13)-Ca(1)-C(15)	82.1(3)
O(4)-Ca(1)-C(15)	109.0(3)
O(3)-Ca(1)-C(15)	76.8(3)
O(6)-Ca(1)-H(1O8)	156.17
O(1)#1-Ca(1)-H(1O8)	96.49(18)
O(8)-Ca(1)-H(1O8)	18.860
O(5)-Ca(1)-H(1O8)	81.69
O(2)-Ca(1)-H(1O8)	52.06
O(13)-Ca(1)-H(1O8)	80.41
O(4)-Ca(1)-H(1O8)	94.44
O(3)-Ca(1)-H(1O8)	126.66
C(15)-Ca(1)-H(1O8)	59.2
O(6)-Ca(1)-H(2O8)	127.84
O(1)#1-Ca(1)-H(2O8)	77.53(18)
O(8)-Ca(1)-H(2O8)	19.812
O(5)-Ca(1)-H(2O8)	90.91
O(2)-Ca(1)-H(2O8)	82.81

O(13)-Ca(1)-H(2O8)	54.16
O(4)-Ca(1)-H(2O8)	115.34
O(3)-Ca(1)-H(2O8)	152.37
C(15)-Ca(1)-H(2O8)	75.7
H(1O8)-Ca(1)-H(2O8)	30.99
O(6)-Ca(1)-H(173)	91.61
O(1)#1-Ca(1)-H(173)	95.75(18)
O(8)-Ca(1)-H(173)	62.87
O(5)-Ca(1)-H(173)	63.83
O(2)-Ca(1)-H(173)	101.68
O(13)-Ca(1)-H(173)	19.257
O(4)-Ca(1)-H(173)	158.73
O(3)-Ca(1)-H(173)	123.30
C(15)-Ca(1)-H(173)	65.0
H(1O8)-Ca(1)-H(173)	64.68
H(2O8)-Ca(1)-H(173)	44.12
O(6)-Ca(1)-H(273)	82.26
O(1)#1-Ca(1)-H(273)	66.36(18)
O(8)-Ca(1)-H(273)	64.36
O(5)-Ca(1)-H(273)	92.42
O(2)-Ca(1)-H(273)	124.75
O(13)-Ca(1)-H(273)	18.229
O(4)-Ca(1)-H(273)	145.16
O(3)-Ca(1)-H(273)	140.58
C(15)-Ca(1)-H(273)	94.8
H(1O8)-Ca(1)-H(273)	75.82
H(2O8)-Ca(1)-H(273)	45.60
H(173)-Ca(1)-H(273)	30.03

Symmetry transformations used to generate equivalent atoms:

#1 -x+1,-y+1,-z+1

Table S10. Anisotropic displacement parameters ($Å^2$x 10^3)for 3d-ca. The anisotropic displacement factor exponent takes the form: $-2\pi^2[h^2a^{*2}U^{11} + ... + 2 h k a^* b^* U^{12}]$

	U^{11}	U^{22}	U^{33}	U^{23}	U^{13}	U^{12}
C(1)	50(9)	250(30)	129(16)	150(20)	46(10)	57(15)
C(2)	73(9)	158(15)	100(11)	103(11)	43(8)	47(10)
C(3)	31(6)	87(9)	52(7)	46(7)	7(5)	9(6)
C(4)	34(6)	67(7)	44(6)	30(6)	4(5)	9(5)
C(5)	29(5)	43(6)	41(6)	20(5)	3(4)	-5(4)
C(6)	42(6)	41(6)	22(5)	2(4)	10(4)	14(5)
C(7)	120(12)	66(9)	35(7)	-7(6)	-2(7)	-17(8)
C(8)	57(8)	39(7)	111(11)	6(7)	25(7)	-8(6)
C(9)	40(7)	48(7)	86(9)	-7(6)	-15(6)	-4(6)
C(10)	24(5)	62(7)	60(7)	23(6)	-4(5)	8(5)
C(11)	72(8)	71(8)	45(6)	29(6)	20(6)	19(7)
C(12)	68(8)	51(7)	59(7)	35(6)	25(6)	17(6)
C(13)	62(7)	30(6)	58(7)	17(5)	12(6)	-13(5)
C(14)	58(7)	44(6)	38(6)	24(5)	4(5)	4(5)
C(15)	88(10)	57(8)	73(9)	12(7)	11(8)	11(7)
C(16)	46(7)	48(7)	62(7)	23(6)	-6(5)	8(5)
C(17)	29(5)	52(7)	35(6)	26(5)	-8(4)	-12(5)
C(18)	36(6)	105(11)	61(8)	63(8)	3(6)	1(7)
C(19)	74(9)	109(12)	113(12)	88(10)	26(9)	9(8)
C(20)	65(11)	170(20)	147(19)	122(17)	26(11)	15(13)
N(1)	45(5)	35(5)	28(4)	10(4)	3(4)	2(4)
O(1)	49(4)	40(4)	34(4)	7(3)	-3(3)	-10(3)
O(2)	62(5)	64(5)	40(4)	25(4)	14(3)	12(4)
O(3)	42(4)	39(4)	64(5)	9(4)	-7(4)	-3(3)
O(4)	60(5)	38(4)	45(4)	24(3)	15(3)	6(3)
O(5)	59(5)	39(4)	60(5)	15(4)	12(4)	4(4)
O(6)	63(5)	46(4)	47(4)	22(4)	16(4)	7(4)
O(7)	58(5)	53(5)	58(5)	33(4)	8(4)	-12(4)
O(8)	44(5)	87(6)	72(5)	20(5)	17(4)	24(4)
O(9)	186(16)	560(40)	420(30)	400(30)	240(20)	240(20)
O(10)	87(8)	162(12)	239(15)	153(12)	21(9)	41(8)
O(11)	340(30)	240(20)	200(20)	96(19)	-20(20)	-50(20)
O(12)	174(12)	146(11)	191(13)	135(11)	81(10)	74(10)

O(13)	102(8)	84(7)	163(11)	43(7)	42(8)	27(6)
O(14)	310(30)	380(30)	360(30)	270(30)	230(20)	280(30)
O(15)	370(40)	740(70)	180(20)	-80(30)	-160(20)	400(50)
O(16)	410(40)	290(30)	540(50)	210(30)	390(40)	130(30)
O(17)	430(40)	66(10)	430(40)	8(15)	190(30)	-79(17)
O(18)	360(40)	280(30)	560(50)	270(30)	320(40)	130(30)
Cl(1)	81(3)	115(3)	101(3)	73(3)	37(2)	35(2)
Cl(2)	46(2)	55(2)	80(2)	28(2)	6(2)	10(1)
Ca(1)	37(1)	36(1)	35(1)	15(1)	0(1)	-1(1)

Table S11. Torsion angles [°] for 3d-ca.

C(20)-C(1)-C(2)-C(3)	-2(2)
C(1)-C(2)-C(3)-C(18)	1.3(18)
C(1)-C(2)-C(3)-C(4)	177.9(11)
C(18)-C(3)-C(4)-C(5)	1.6(14)
C(2)-C(3)-C(4)-C(5)	-175.0(10)
C(3)-C(4)-C(5)-C(17)	-4.9(14)
C(3)-C(4)-C(5)-C(6)	-178.7(9)
C(4)-C(5)-C(6)-O(1)	71.5(13)
C(17)-C(5)-C(6)-O(1)	-102.3(10)
C(4)-C(5)-C(6)-N(1)	-111.6(10)
C(17)-C(5)-C(6)-N(1)	74.6(11)
O(5)-C(8)-C(9)-O(3)	-50.4(15)
O(2)-C(11)-C(12)-O(4)	-56.4(11)
O(4)-C(13)-C(14)-N(1)	57.0(11)
O(2)-C(7)-C(15)-O(5)	43.0(17)
O(2)-C(7)-C(15)-Ca(1)	8.3(10)
N(1)-C(10)-C(16)-O(3)	67.4(12)
C(4)-C(5)-C(17)-O(6)	-173.8(9)
C(6)-C(5)-C(17)-O(6)	0.4(13)
C(4)-C(5)-C(17)-O(7)	3.9(13)
C(6)-C(5)-C(17)-O(7)	178.2(8)
C(2)-C(3)-C(18)-O(7)	179.7(10)
C(4)-C(3)-C(18)-O(7)	2.8(15)
C(2)-C(3)-C(18)-C(19)	-0.2(17)
C(4)-C(3)-C(18)-C(19)	-177.0(10)
O(7)-C(18)-C(19)-C(20)	179.7(12)
C(3)-C(18)-C(19)-C(20)	-0.4(19)
C(2)-C(1)-C(20)-C(19)	1(3)
C(18)-C(19)-C(20)-C(1)	0(3)
O(1)-C(6)-N(1)-C(10)	-169.9(9)
C(5)-C(6)-N(1)-C(10)	13.3(13)
O(1)-C(6)-N(1)-C(14)	-3.4(14)
C(5)-C(6)-N(1)-C(14)	179.8(8)
C(16)-C(10)-N(1)-C(6)	-129.3(10)
C(16)-C(10)-N(1)-C(14)	63.9(12)
C(13)-C(14)-N(1)-C(6)	74.9(11)

C(13)-C(14)-N(1)-C(10)	-117.8(10)
N(1)-C(6)-O(1)-Ca(1)#1	133.8(19)
C(5)-C(6)-O(1)-Ca(1)#1	-49(3)
C(15)-C(7)-O(2)-C(11)	-165.4(11)
C(15)-C(7)-O(2)-Ca(1)	-12.5(15)
C(12)-C(11)-O(2)-C(7)	-176.1(10)
C(12)-C(11)-O(2)-Ca(1)	30.8(11)
C(8)-C(9)-O(3)-C(16)	-172.4(10)
C(8)-C(9)-O(3)-Ca(1)	27.0(13)
C(10)-C(16)-O(3)-C(9)	135.3(10)
C(10)-C(16)-O(3)-Ca(1)	-67.8(11)
C(14)-C(13)-O(4)-C(12)	129.5(9)
C(14)-C(13)-O(4)-Ca(1)	-91.1(9)
C(11)-C(12)-O(4)-C(13)	-159.1(9)
C(11)-C(12)-O(4)-Ca(1)	56.7(9)
C(7)-C(15)-O(5)-C(8)	87.3(14)
Ca(1)-C(15)-O(5)-C(8)	140.9(11)
C(7)-C(15)-O(5)-Ca(1)	-53.6(13)
C(9)-C(8)-O(5)-C(15)	-86.5(13)
C(9)-C(8)-O(5)-Ca(1)	52.0(13)
O(7)-C(17)-O(6)-Ca(1)	167.7(14)
C(5)-C(17)-O(6)-Ca(1)	-14(2)
O(6)-C(17)-O(7)-C(18)	178.2(9)
C(5)-C(17)-O(7)-C(18)	0.3(13)
C(3)-C(18)-O(7)-C(17)	-3.8(14)
C(19)-C(18)-O(7)-C(17)	176.1(9)

Symmetry transformations used to generate equivalent atoms:

#1 -x+1,-y+1,-z+1

Table S12. Hydrogen bonds for 3d-ca [Å and °].

D-H...A	d(D-H)	d(H...A)	d(D...A)	<(DHA)
C(13)-H(11)...O(1)	0.97	2.62	3.128(12)	113.1
C(13)-H(11)...O(1)#1	0.97	2.56	3.278(13)	131.1
C(13)-H(11)...O(1)	0.97	2.62	3.128(12)	113.1
C(13)-H(11)...O(1)#1	0.97	2.56	3.278(13)	131.1
C(10)-H(27)...O(17)#2	0.97	2.44	3.28(2)	144.1
C(13)-H(11)...O(1)	0.97	2.62	3.128(12)	113.1
C(13)-H(11)...O(1)#1	0.97	2.56	3.278(13)	131.1
C(14)-H(10)...O(12)#3	0.97	2.57	3.412(16)	145.9
C(15)-H(14)...O(15)#4	0.97	2.60	3.41(3)	142.0
O(8)-H(1O8)...O(2)	0.8495	2.239	2.759	119.52
O(8)-H(1O8)...O(9)	0.8495	2.401	2.822	111.2
O(8)-H(2O8)...O(13)	0.8492	2.2703	2.9473	136.809
O(18)-H(132)...O(16)	0.8511	2.60	3.33	144.7

Symmetry transformations used to generate equivalent atoms:

#1 -x+1,-y+1,-z+1 #2 x-1,y,z #3 -x+1,-y+1,-z

#4 -x+1,-y,-z
